# The parasitic worm product ES-62 promotes health- and life-span in a high calorie diet-accelerated mouse model of ageing

**DOI:** 10.1371/journal.ppat.1008391

**Published:** 2020-03-12

**Authors:** Jenny Crowe, Felicity E. Lumb, James Doonan, Margaux Broussard, Anuradha Tarafdar, Miguel A. Pineda, Carmen Landabaso, Lorna Mulvey, Paul A. Hoskisson, Simon A. Babayan, Colin Selman, William Harnett, Margaret M. Harnett

**Affiliations:** 1 Institute of Infection, Immunity and Inflammation, University of Glasgow, Glasgow, United Kingdom; 2 Strathclyde Institute of Pharmacy and Biomedical Sciences, University of Strathclyde, Glasgow, United Kingdom; 3 Glasgow Ageing Research Network (GARNER), Institute of Biodiversity, Animal Health and Comparative Medicine, University of Glasgow, Glasgow, United Kingdom; University of Medicine & Dentistry New Jersey, UNITED STATES

## Abstract

Improvements in hygiene and health management have driven significant increases in human lifespan over the last 50 years. Frustratingly however, this extension of lifespan has not been matched by equivalent improvements in late-life health, not least due to the global pandemic in type-2 diabetes, obesity and cardiovascular disease, all ageing-associated conditions exacerbated and accelerated by widespread adoption of the high calorie Western diet (HCD). Recently, evidence has begun to emerge that parasitic worm infection might protect against such ageing-associated co-morbidities, as a serendipitous side-effect of their evolution of pro-survival, anti-inflammatory mechanisms. As a novel therapeutic strategy, we have therefore investigated the potential of ES-62, an anti-inflammatory secreted product of the filarial nematode *Acanthocheilonema viteae*, to improve healthspan (the period of life before diseases of ageing appear) by targeting the chronic inflammation that drives metabolic dysregulation underpinning ageing-induced ill-health. We administered ES-62 subcutaneously (at a dose of 1 μg/week) to C57BL/6J mice undergoing HCD-accelerated ageing throughout their lifespan, while subjecting the animals to analysis of 120 immunometabolic responses at various time-points. ES-62 improved a number of inflammatory parameters, but markedly, a range of pathophysiological, metabolic and microbiome parameters of ageing were also successfully targeted. Notably, ES-62-mediated promotion of healthspan in male and female HCD-mice was associated with different mechanisms and reflecting this, machine learning modelling identified sex-specific signatures predictive of ES-62 action against HCD-accelerated ageing. Remarkably, ES-62 substantially increased the median survival of male HCD-mice. This was not the case with female animals and unexpectedly, this difference between the two sexes could not be explained in terms of suppression of the chronic inflammation driving ageing, as ES-62 tended to be more effective in reducing this in female mice. Rather, the difference appeared to be associated with ES-62’s additional ability to preferentially promote a healthier gut-metabolic tissue axis in male animals.

## Author summary

Infection with parasitic worms is increasingly considered to protect against chronic inflammatory disorders, including obesity, type-2 diabetes and cardiovascular disease. Although previously associated with old age, these ailments are ever more prevalent even in young people, due to the global adoption of the “Western” lifestyle and high calorie diet (HCD; high sugar and high fat). Rather than developing live worm therapies, our approach focuses on the potential of ES-62, a molecule secreted by the parasitic worm *Acanthocheilonema viteae* that we have previously shown to protect against inflammatory conditions like allergic asthma and arthritis in mice. We found that a small weekly dose of ES-62 promoted late-life wellbeing in HCD-fed mice of both sexes and substantially extended the life of obese male mice (by over 70 days; >10%). Machine learning modelling of its impact on the complex immunometabolic networks underpinning obesity showed that health-promoting effects of ES-62 could not simply be explained by its anti-inflammatory actions but encompassed protection against adipose, liver and gut pathology. The target signatures identified may thus represent a starting point for developing novel ES-62-based approaches to improving health during ageing and increasing lifespan in humans.

## Introduction

Adoption of the modern Western high calorie diet (HCD; high fat and high sugar) has significantly contributed to the global major public health problems of metabolic syndrome and resultant obesity, type-2 diabetes and cardiovascular disease; ageing-associated comorbidities that impact on both healthspan and lifespan [1,2]. Interestingly, epidemiological data suggest that such HCD-associated diseases are rising fastest in in the developing world [2,3], regions where parasitic worms (helminths) and other infectious agents used to be endemic but where they are now being eradicated [4]. Helminths promote their survival by releasing excretory-secretory (ES) products that, by dampening inflammation and promoting tissue repair, act to prevent worm expulsion but also limit host pathology [4,5]. Thus, the relatively rapid eradication of these parasites appears to have resulted in hyper-active host immune systems, characterised by chronic low-grade inflammation that may (further) contribute to development of obesity and associated metabolic syndrome co-morbidities as well as their reciprocal risk factors, allergic and autoimmune inflammatory disorders [6], in developing and urbanised countries [4,5]. Whilst this has questioned the wisdom of current mass parasite eradication programs, it has emphasised the potential of utilising worm infections or ES products to treat a wide range of non-communicable diseases characterised by chronic inflammation [7–9].

We have previously shown that a single, defined ES protein, ES-62, can resolve chronic inflammation by normalising aberrant MyD88 signalling to homeostatically restore immunoregulation, irrespective of the inflammatory phenotype [4,5,10–12]. MyD88 is increasingly recognized not only as a key innate immune system receptor transducer but also as an integrator of dysregulated inflammatory and metabolic pathways [7,13–15]. Indeed, adoption of a HCD induces reprogramming of innate immune responses, with the resulting chronic TLR4/MyD88 signalling [16] playing critical roles in promoting generation of pro-inflammatory (M1-like CD11c^+^) macrophages, glucose intolerance, β-cell failure and consequent inflammation of metabolic (adipose, pancreas and liver) tissues [16,17]. Critically, these HCD-TLR4/MyD88-induced effects are exacerbated by ageing [18]. Thus, based on ES-62’s targeting of MyD88 and demonstrable ability to protect against chronic inflammatory allergic and autoimmune conditions [4,5,10–12], we investigated whether it could safeguard against the impact of HCD-associated ageing in C57BL/6J mice. Our approach of combining longitudinal (survival) and cross-sectional (intervention) studies has revealed that ES-62 improves healthspan in both male and female HCD-fed mice and substantially extends median lifespan of male HCD-fed mice. Unexpectedly, ageing male and female mice exhibited quite distinct immunometabolic responses to HCD and perhaps reflecting this sexual dimorphism, ES-62 displayed certain sex-specific mechanisms of protection. Further important insight into the mechanisms by which ES-62 protects against the consequences of HCD-accelerated ageing to promote healthspan in male and female mice was provided by machine learning modelling of the multidimensional data sets encompassing 120 pathophysiological parameters of HCD-accelerated ageing generated in the study. Critically, this strategy identified sex-specific immunological and metabolic healthspan signatures predictive of ES-62 action. Specifically, and consistent with the current proposed key role of chronic low-grade inflammation in ageing, these signatures indicated that increased healthspan in female HCD-fed mice predominantly reflected ES-62's well characterised anti-inflammatory activity. Unexpectedly, therefore, improved healthspan and consequently extended lifespan in male HCD mice could not be explained solely by its restoration of immunoregulation but rather, these signatures highlighted that ES-62 additionally acted to normalize the gut microbiota to maintain health of the gut-metabolic tissue axis.

## Results

### ES-62 extends median lifespan in male HCD-fed C57BL/6J mice

**Fig 1:**
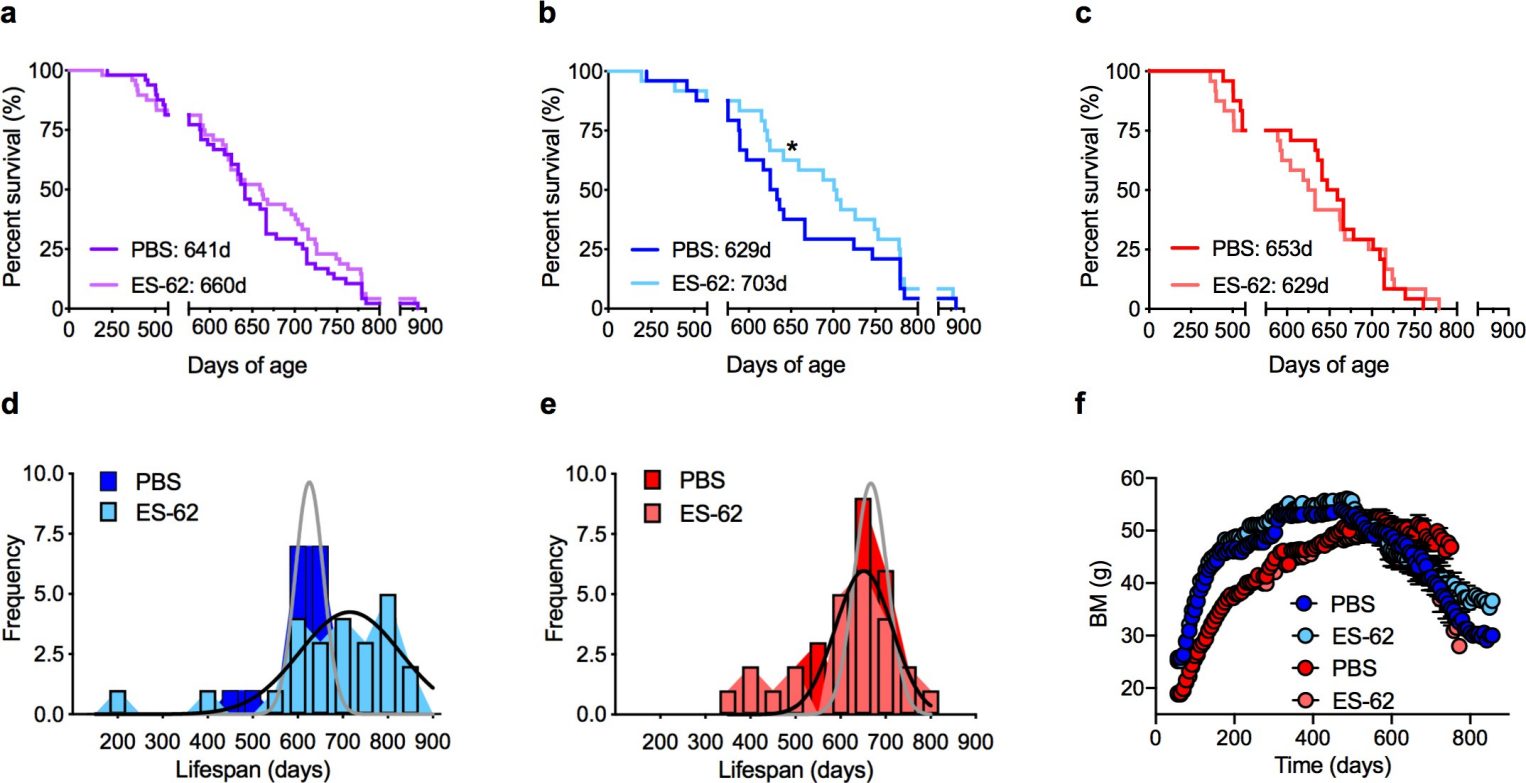
ES-62 extends lifespan in male HCD-fed C57BL/6J mice. Kaplan-Meier survival curves (a-c) for HCD-fed mice treated either with PBS or ES-62 were analysed as (a) combined sex cohorts (PBS, n = 48; ES-62, n = 48); (b) male mice (PBS, n = 24; ES-62, n = 24, *p<0.05) only and (c) female mice (PBS, n = 24; ES-62, n = 24) only. Median survival values for the relevant groups are shown as inserts. The data for the male and female cohorts are also presented as the frequency distribution of lifespan (d, male; e, female). Longitudinal analysis of body mass (BM; f) measurements was undertaken where data are presented as mean values ± SEM of the individual mice (Male, blue symbols; Female, red symbols) surviving at the indicated time-points.

ES-62 did not significantly increase the median lifespan of a combined sex cohort of HCD-fed C57BL/6J mice (Fig 1A). However, and consistent with previous findings that interventions that impact on health- and lifespan often exhibit sexual dimorphism [19–21], Cox regression analysis (S1 Table) revealed significant sex differences. Thus, ES-62 substantially extended the median lifespan of male (PBS, 629; ES-62, 703 days), but not female (PBS, 653; ES-62, 629 days) HCD-mice (Fig 1B–1E). Post-mortem analysis (S2 Table) did not reveal any clear protection against life-threatening pathologies associated with life-long exposure to an energy-rich diet, with typically, many of the mice within our lifespan study exhibiting evidence of liver tumours irrespective of sex or treatment with ES-62.

Low body mass (BM), particularly in early life (<d150), is associated with longevity in genetically heterogeneous mice, fed a normal chow diet [22]. Notably therefore, our correlative analysis of BM at d116, 160, 340 and 500 demonstrated that this association was not recapitulated in any of our HCD-fed cohorts (S1 Fig): indeed, the extension of life-span in male ES-62-HCD-fed mice did not reflect prevention of HCD-increased BM as ES-62 did not significantly reduce either peak BM (Male: PBS, 56.20 ± 0.93; ES-62, 57.22 ± 1.10 g; Female: PBS, 53.19 ± 1.32, ES-62, 50.77 ± 1.17 g) or BM over the course of weight gain (Fig 1F). Reflecting this, food intake was not altered by ES-62 in either male or female HCD-fed mice (S2A Fig). Of note, although BM declined sharply after reaching maximal levels in HCD (PBS and ES-62)-mice, ES-62 appeared to protect against the weight loss associated with late ageing (>720-day old mice) in surviving male, but not female, HCD-fed mice (Fig 1F). Typically, lean mass declines whilst fat mass increases with age, and so this may suggest that ES-62 acts to preserve lean mass and prevent sarcopenia. However, there was no corresponding protection afforded by ES-62 against decline in grip strength, one of the indicators of age-associated frailty (S2B Fig).

To address identifying the protective actions of ES-62 in the model we examined its impact on key HCD-target tissues (adipose, liver and gut) and their associated dysregulated functional responses in a series of cross-sectional studies (at d160, d340 and d500) comparing male and female HCD-fed mice with young (day 56) and aged-matched chow-fed control cohorts. Longitudinal analysis showed that mice in the cross-sectional cohorts exhibited very similar kinetics of BM gain to those in the longevity study (S2C and S2D Fig), with scrutiny of their mean BM values and organ sizes at cull independently confirming that ES-62 did not exert any substantial effect on HCD-induced obesity, *per se* (S2E and S2F Fig).

### ES-62 protects against visceral adipose tissue dysfunction in male HCD-fed mice

**Fig 2:**
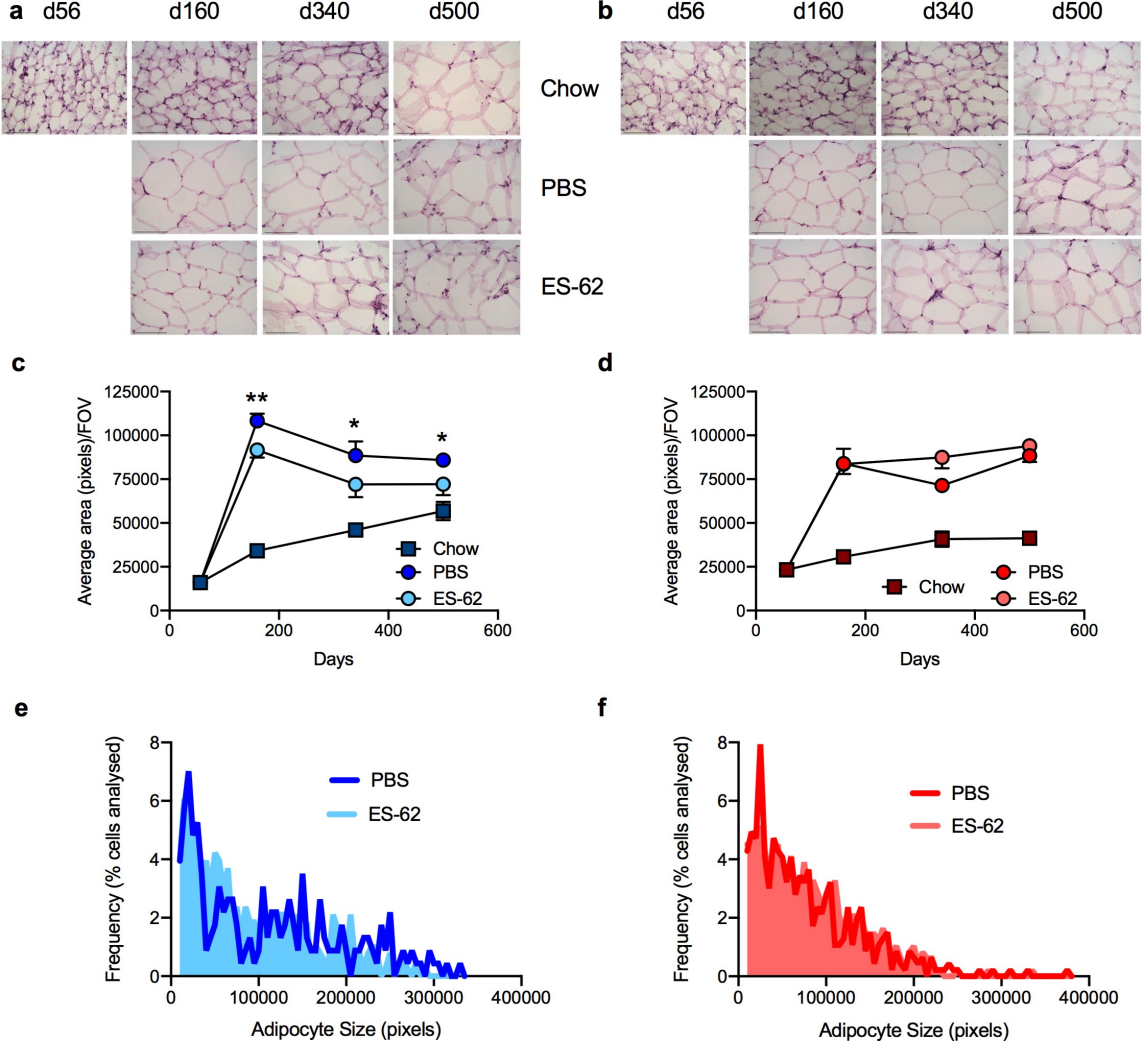
ES-62 ameliorates gonadal adipocyte hypertrophy in male HCD-fed mice. Representative images (scale bar 100 μm) of gonadal fat from male (a) and female (b) chow- and HCD- (PBS or ES-62-treated) mice stained with H & E and resultant quantitative analysis of average adipocyte size where data are presented as the mean values ± SEM, for individual male (c) and female (d) mice at each time point and the values for each mouse are means derived from n = 3 replicate analyses. Male cohort sizes: chow—d56, n = 3; d160, n = 4; d340, n = 4; d500, n = 2; HCD-PBS—d160, n = 5; d340, n = 4; d500, n = 6; HCD-ES-62—d160, n = 5; d340, n = 4; d500, n = 5 and female cohort sizes: chow—d56, n = 5; d160, n = 4; d340, n = 3; d500, n = 4; HCD-PBS—d160, n = 5; d340, n = 5; d500, n = 6; HCD-ES-62—d160, n = 6; d340, n = 4; d500, n = 4. For clarity, only significant differences between the HCD-PBS and HCD-ES-62 cohorts are shown on the figures, where significance is denoted by *p < 0.05 and **p < 0.01. The ES-62 reduction of adipocyte hypertrophy in male, but not female, mice is also illustrated by analysis of the frequency of individual adipocyte sizes (pixels) in HCD-PBS and HCD-ES-62 tissue. Here, data for all the images analysed are presented as the frequency of adipocytes of the indicated size expressed as the % of total adipocytes analysed: Mann-Whitney analysis of the mean rank values and Kolmogorov analysis both showed the distribution of adipocyte sizes for HCD-ES-62 to be significantly different (p<0.05) from that of HCD-PBS for male (e), but not female (f), mice.

As expected under conditions of obesity, male and female mice fed a HCD exhibited pronounced adipocyte hypertrophy in their gonadal visceral fat deposits (evidenced by their increased average size and reduced cell number/field of view (FOV) in tissue sections; Fig 2A–2D) relative to the slow progressive increase in adipocyte size reflective of the loss of metabolic regulation occurring during ageing [23], observed in the chow-fed cohorts (Fig 2A–2D). With respect to the HCD-mice, ES-62 was able to reduce the hypertrophy observed in gonadal visceral fat depots in male, but not female, mice (Fig 2A–2F).

**Fig 3:**
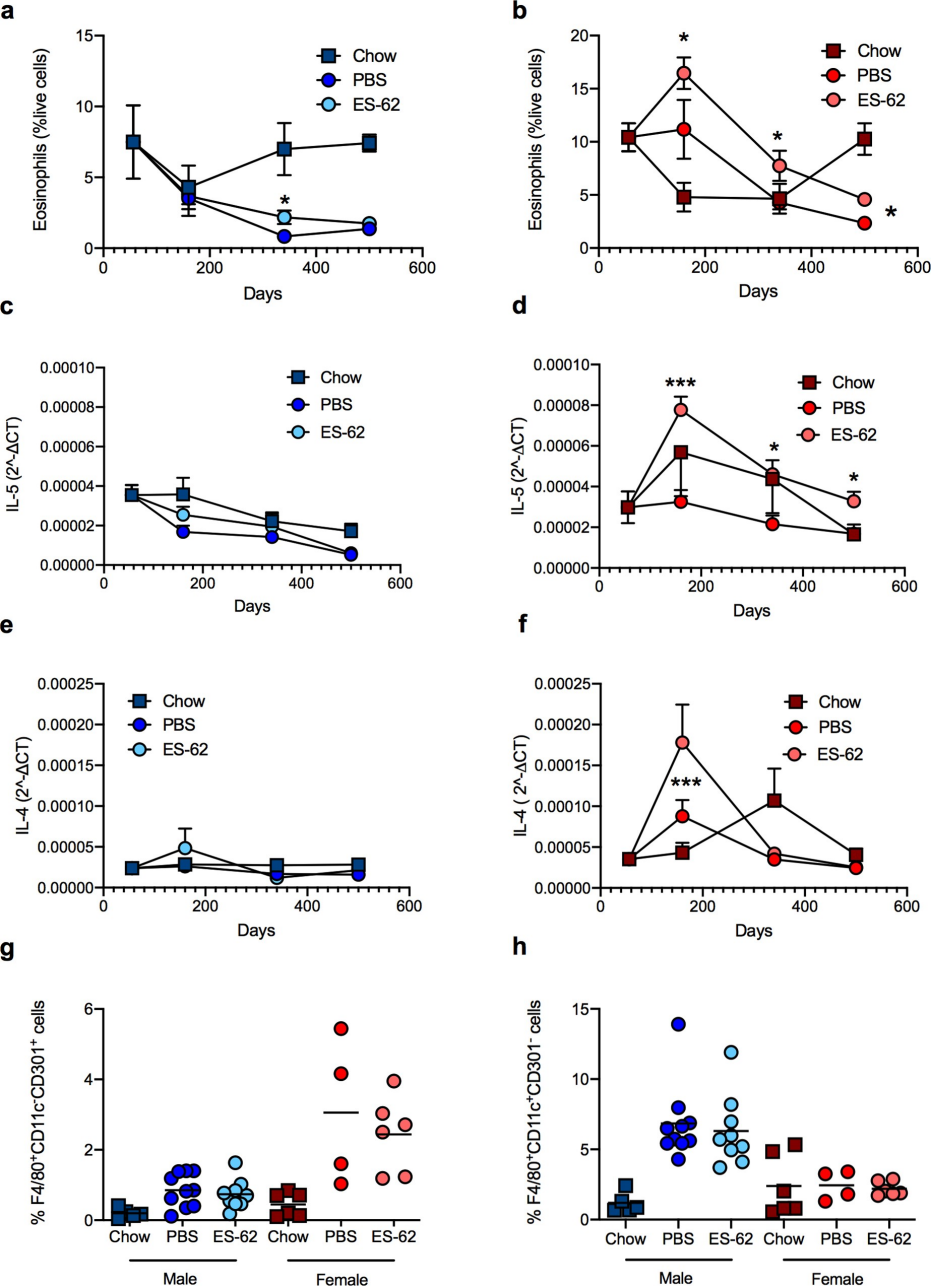
ES-62 modulation of eosinophil levels in gonadal fat of HCD-fed mice. The levels of SiglecF^+^ eosinophils (% SVF cells) from gonadal fat of chow- and HCD- (PBS- or ES-62-treated) mice are presented as the mean values ± SEM at each time point where male (a) cohort sizes are: chow—d56, n = 5; d160, n = 3; d340, n = 6; d500, n = 5; HCD-PBS—d160, n = 10; d340, n = 11; d500, n = 6; HCD-ES-62—d160, n = 9; d340, n = 12; d500, n = 6 and female (b) cohort sizes are: chow d56—n = 6; d160, n = 6; d340, n = 6; d500, n = 5; HCD-PBS—d160, n = 4; d340, n = 11; d500, n = 6; HCD-ES-62—d160, n = 6; d340, n = 12; d500, n = 6. qRT-PCR analysis of IL-5 and IL-4 mRNA expression in gonadal fat from chow- and HCD-fed (PBS- or ES-62-treated) mice is presented (c-f) as mean 2^ΔCT values ± SEM of individual mice and the values for each mouse are means of n = 3 replicate analyses. Male cohort sizes: chow—d56, n = 6; d160, n = 5; d340, n = 6; d500, n = 5; HCD-PBS—d160, n = 10; d340, n = 11; d500, n = 6; HCD-ES-62—d160, n = 9 (IL-5), n = 10 (IL-4); d340, n = 12; d500, n = 6. Female cohort sizes: chow—d56, n = 6; d160, n = 5; d340, n = 5; d500, n = 5; HCD-PBS—d160, n = 8 (IL-4), n = 9 (IL-5) d340, n = 11; d500, n = 6; HCD-ES-62—d160, n = 9 (IL-4), n = 10 (IL-5); d340, n = 11; d500, n = 6. The levels of F4/80^+^CD11c^-^CD301^+^ (g) and F4/80^+^CD11c^+^Cd301^-^ (h) macrophages in gonadal fat in male and female chow- and HCD- (PBS- or ES-62-treated) mice in the d160 cohorts are shown. For clarity, only significant differences between the HCD-PBS and HCD-ES-62 cohorts are shown on the figures, where significance is denoted by *p < 0.05 and ***p < 0.001.

Eosinophils have been proposed to maintain adipocyte health by promoting, in an IL-4/5-dependent manner, M2-like macrophages that act to preserve metabolic function and prevent adiposity and systemic insulin resistance [24,25]. Consistent with this, whilst their levels in gonadal fat tissues (Fig 3A) remain relatively constant throughout ageing in male chow-fed mice, they are progressively reduced by HCD diet. Overall, ES-62 treatment provides marginal (albeit significant at d340) protection against this decline. Perhaps surprisingly therefore, given the associated adipocyte hypertrophy, the levels of eosinophils were even higher in such visceral fat from female HCD-PBS mice at d160 than those found in young lean female mice (Fig 3B). Moreover, despite its failure to suppress hypertrophy, eosinophils in the female HCD cohorts were further boosted throughout the time-course by treatment with ES-62, with levels only declining to below those observed in chow mice between d340-500 (Fig 3B). Supporting these contrasting findings across the two sexes, the eosinophil profiles were broadly mirrored by the pattern of type-2 cytokine mRNA expression in visceral fat: thus, in male mice, ES-62 again exhibited marginal effects, whilst in female mice, the HCD-ES-62 cohort exhibited the highest expression of IL-5 (Fig 3C and 3D). Moreover, ES-62 strongly upregulated IL-4 expression in the gonadal fat of the female, but not male, d160 cohort of HCD-fed mice (Fig 3E and 3F). Reflecting the enhanced levels of eosinophils and type-2 cytokines (IL-4 and IL-5) achieved, there is a greater increase in M2 (CD11c^-^CD301b^+^)-like macrophages in gonadal fat tissue at d160, in both PBS- and ES-62-treated female HCD mice than in the male HCD cohorts, which instead displayed a strong rise in M1 (CD11c^+^CD301b)-like macrophages (Fig 3G and 3H). Collectively therefore, these data identified a perhaps surprising disconnect between the ability of ES-62 to promote type-2 responses and a leaner phenotype in gonadal fat.

To address determining whether this sex bias and the disconnect between type-2 responses and adipose tissue health was restricted to gonadal fat, we also investigated whether ES-62 similarly modulated adipose tissue dysfunction in the retroperitoneal visceral fat beds. Here we again found that HCD accelerated the ageing-associated adipocyte hypertrophy observed in both cohorts of chow-fed mice, although in this tissue the male chow-fed mice also exhibited substantial increases in the size of the adipocytes, even at the d160 time-point (S3A–S3D Fig). Although failing to significantly inhibit HCD-induced adipocyte hypertrophy, ES-62 again exhibited some degree of protection against HCD-induced pathology in male, but not female, mice, with the adipocyte size in HCD-ES-62 mice being not significantly different from that in the male chow cohorts (S3A–S3D Fig). However, ES-62 treatment did significantly restrain the HCD-induced suppression of eosinophils, IL-5 and M2-macrophage expression in retroperitoneal fat tissue from male HCD-fed mice whereas no significant increase in any of the eosinophil, cytokine (IL-5 or IL-4) or M2-like macrophage responses could be detected in the female HCD-ES-62 mice (S3E–S3K Fig). Moreover, despite exhibiting substantial hypertrophy of retroperitoneal adipocytes between d160-d500, there were no significant differences in eosinophil levels at any of the time points examined in male chow-fed mice (S3E–S3K Fig). Thus, once again, our data identify a disconnect between type-2 responses and visceral adipose tissue health in this obesity-accelerated ageing mode (Figs 2 and 3; S3 Fig)

### ES-62 does not prevent HCD-induced Insulin Resistance in either male or female mice

Adipocyte hypertrophy has been reported to lead to insulin resistance and liver fibrosis via dysregulation of adipokines, typically evidenced by elevated levels of leptin and suppression of adiponectin production in obesity [26–28]. Reflecting this, serum levels of leptin were high in male and female HCD-PBS mice of the d500 cohort, findings consistent with induction of leptin resistance: these rises were attenuated in ES-62-treated mice of both sexes, where leptin levels were not significantly different from those observed in age-matched chow-fed animals (S4A and S4B Fig). By contrast, ES-62 did not prevent the HCD-suppression of the rise in serum adiponectin observed with age in lean male and female mice (S4C and S4D Fig). Consistent with these adipokine effects contributing to insulin resistance (IR), serum insulin levels were substantially elevated in HCD-fed mice (S4E and S4F Fig). Reflecting the HCD-induced increased IR, analysis of pancreatic β-cell function (S5A–S5H Fig) revealed that it was accompanied by a compensatory hyper-production of insulin and glucagon, followed by islet death typical of the pancreatic failure associated with established Type-2 diabetes in humans [26]. ES-62 had no significant protective effect on the progressively developing IR, serum insulin levels and pancreatic β-cell function (S4 Fig; S5 Fig) nor on the outcome of the Homeostatic Model Assessment of Insulin Resistance (HOMA-IR) test (S6A and S6B Fig).

Reflecting these pathological changes, increased fasting blood glucose levels and aberrant glucose clearance (as indicated by their glucose tolerance test [GTT] responses) were evident in HCD- but not chow-fed mice from d160 onwards (S6C–S6H Fig). ES-62 exhibited only marginal effects, slightly slowing the age-associated rise in fasting glucose levels observed in female HCD-PBS mice (S6D Fig). Unlike the strong impairment in glucose clearance widely documented for mice acutely fed a HCD (up to ~10 weeks), and likely reflecting the remodelling of glucose handling reported to result from the increased adipose tissue providing novel glucose sinks in mice fed a chronic HCD [29], whilst female HCD-mice exhibited only a slight delay in glucose clearance, male HCD-mice showed apparently substantially improved glucose handling relative to their chow-controls. In any case, as ES-62 did not significantly modulate glucose handling in (either male or female) HCD-fed mice (S6 Fig) throughout the cross-sectional analysis, its lifespan-promoting effects appear to be unrelated to the improved glucose tolerance previously observed in acute, short-term mouse models of obesity following infection with helminths, or treatment with their products [30–34].

### ES-62 reduces liver fibrosis in both male and female HCD-fed mice

**Fig 4:**
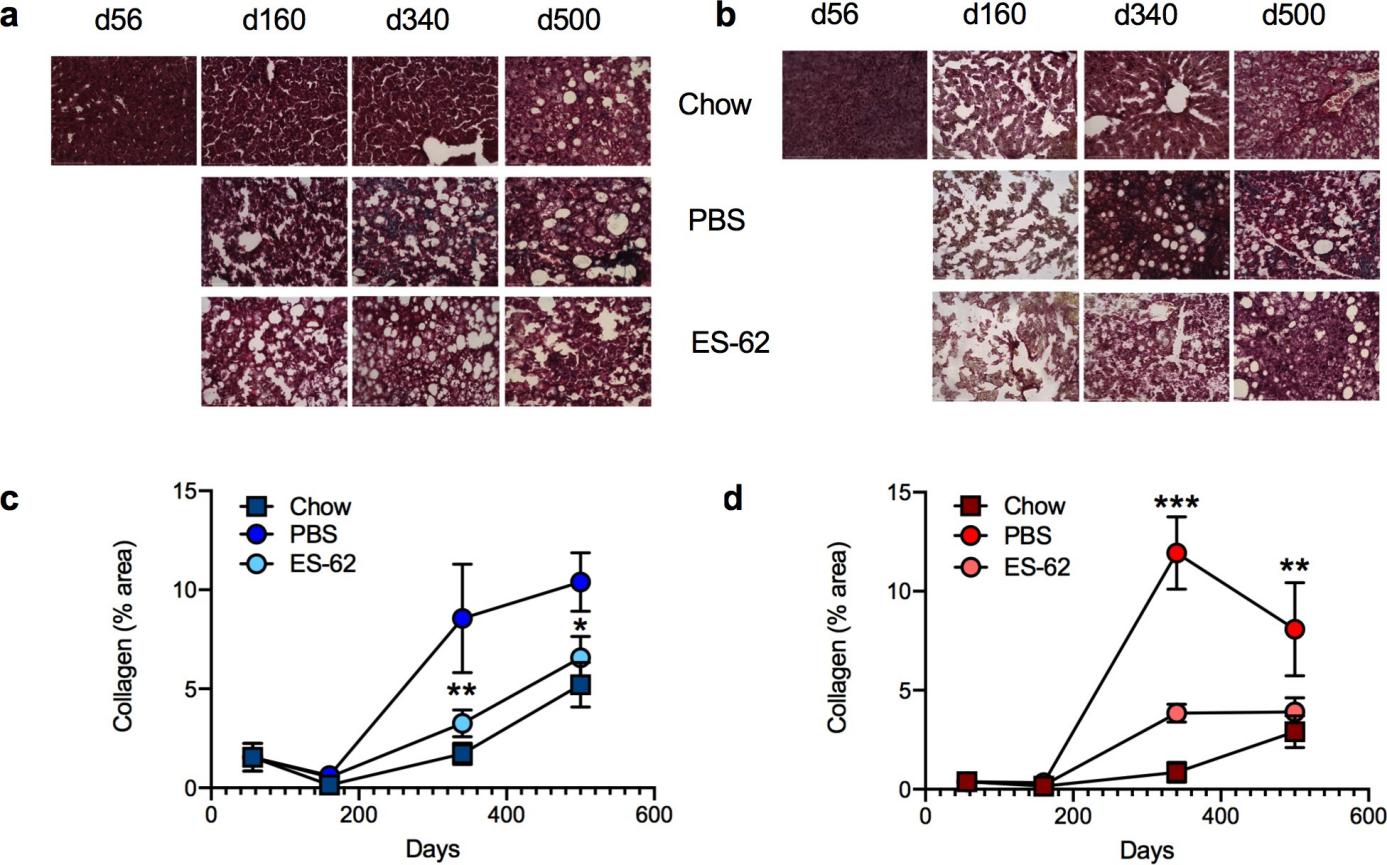
Effect of ES-62 on liver fibrosis in HCD-fed mice. Representative images (scale bar 100 μm) of liver from male (a) and female (b) mice stained with Gömöri’s Trichrome: quantitative analysis of collagen deposition is presented as mean (of triplicate analyses) values ± SEM, where n = 5–6 individual male (c) and female (d) mice. Significant differences between the HCD-PBS and HCD-ES-62 cohorts are shown where *p < 0.05, **p < 0.01 and ***p < 0.001.

HCD-mediated disruption of adipocyte health and induction of IR consequently impacts on liver function [35] leading to liver steatosis, fibrosis and cancer. Consistent with this, there was a dramatic but transient increase in the levels of fatty liver in both male and female HCD-fed mice that then appeared to resolve/remodel, resulting in slightly elevated levels of fatty liver being exhibited by the chow and HCD-fed mice in the d500 cohorts (S7A–S7D Fig). Consequent to the spikes in liver steatosis incidence, we observed substantial acceleration and enhancement of age-related liver fibrosis (as evidenced by collagen deposition) in both male and female HCD-PBS mice. Critically, exposure to ES-62 ameliorated HCD-induced liver fibrosis in both sexes, maintaining the slow kinetics and low incidence of age-related liver fibrosis observed in the chow-fed controls (Fig 4A–4D).

To address identifying the mechanisms involved, we assessed the impact of ES-62 on a number of parameters (mitochondrial dysfunction, REDOX imbalance and inflammation) implicated in liver pathogenesis and HCD-accelerated ageing. As expected, HCD and ageing induced profound impairment of respiration rates within isolated liver mitochondria of both male and female mice over time (S7E–S7J Fig). ES-62 partially rescued the HCD-induced impairment in mitochondrial function in male mice at d160, but its effects in female mice were much less pronounced and beneficial effects were not observed in the older HCD-fed cohorts (S7 Fig). Likewise, ES-62 did not protect against the HCD-accelerated decrease in cytochrome C expression (S8A and S8B Fig) previously associated with age-dependent decreases in mitochondrial respiratory capacity [36] nor did it substantially impact on a variety of parameters associated with the REDOX balance, namely HMOX1 expression and superoxide dismutase (SOD) and protein carbonylation activities (S8C–S8H Fig). Perhaps more surprisingly, given its anti-inflammatory activities, the effects of ES-62 were marginal, particularly low in male mice, on the transient spikes in inflammatory cytokine (IL-1β and IL-18) and associated NLRP3 expression paralleling the dramatic increase and remodelling of fat deposition within livers from HCD-fed mice (S9A–S9F Fig).

Thus, in the absence of organ-specific mechanisms to explain ES-62 protection against metabolic tissue dysregulation, we next analysed the effect of ES-62 on gut health, as HCD-induced gut inflammation, loss of barrier integrity and associated dysbiosis of the microbiome are key factors linked not only to the development of visceral fat inflammation, IR and obesity [37,38] but also to the acceleration of ageing and shortening of lifespan [39,40].

### ES-62 promotes gut health in male HCD-fed mice

**Fig 5:**
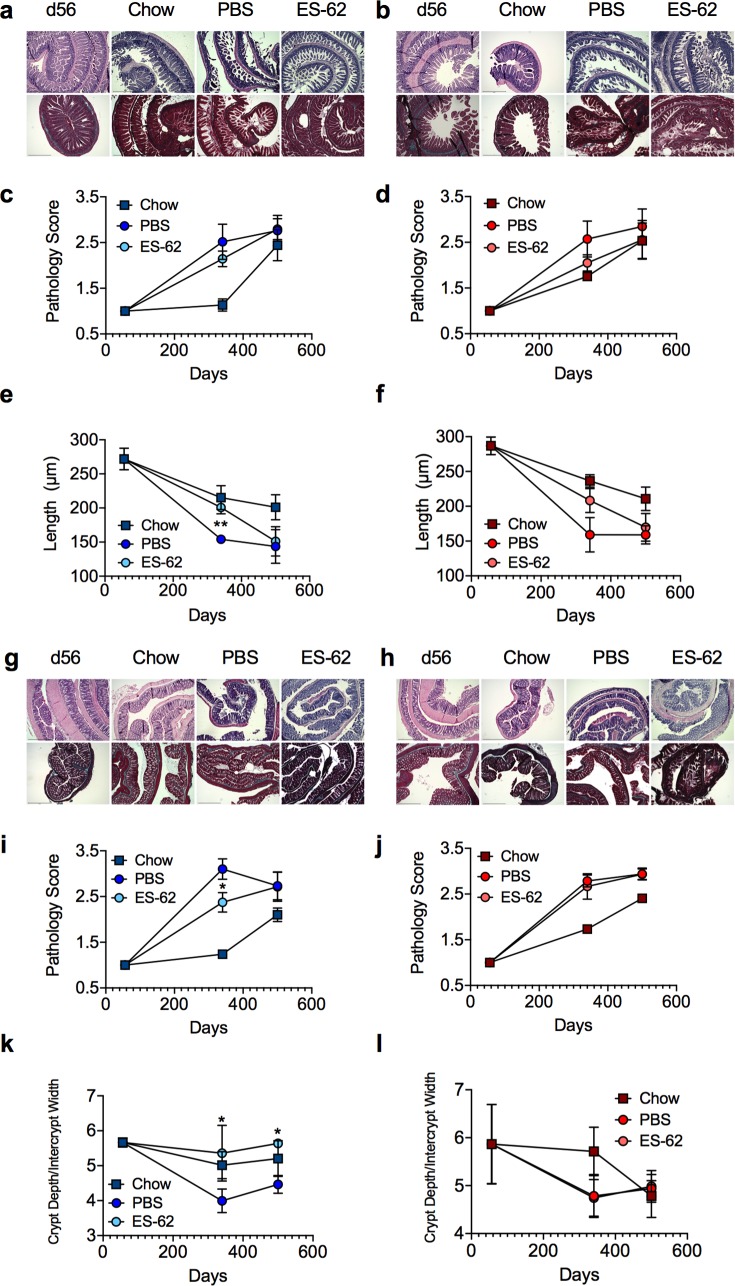
ES-62 protects against gut pathology in HCD-fed mice. Representative images (scale bar 500 μm) of ileum tissue from male (a) and female (b) d56 (chow-fed) and d340 chow- and HCD- (PBS- or ES-62-treated) mice stained with H & E (upper panels) and Gömöri’s Trichrome (lower panels) and resultant pathology scoring (c, d) and quantitative analysis of ileum villus length (e, f) are shown. Data are presented as the mean values ± SEM where n = 4–6 individual male (a, c, e) and female (b, d, f) mice at each time point and the values for each mouse are means derived from n = 3 replicate analyses. Representative images (scale bar 500 μm) of colon tissue from male (g) and female (h) d56 (chow-fed) and d340 chow- and HCD- (PBS- or ES-62-treated) mice stained with H & E (upper panels) and Gömöri’s Trichrome (lower panels) and resultant pathology scoring (i, j) and quantitative analysis of the ratio of crypt depth:intercrypt width (k, l) are shown. Data are presented as the mean values ± SEM where n = 4–6 individual male (g, i, k) and female (h, j, l) mice at each time point and the values for each mouse are means derived from n = 3 replicate analyses. For clarity, only significant differences between the HCD-PBS and HCD-ES-62 cohorts are shown on the figures, where significance is denoted by *p < 0.05 and **p < 0.01.

**Fig 6:**
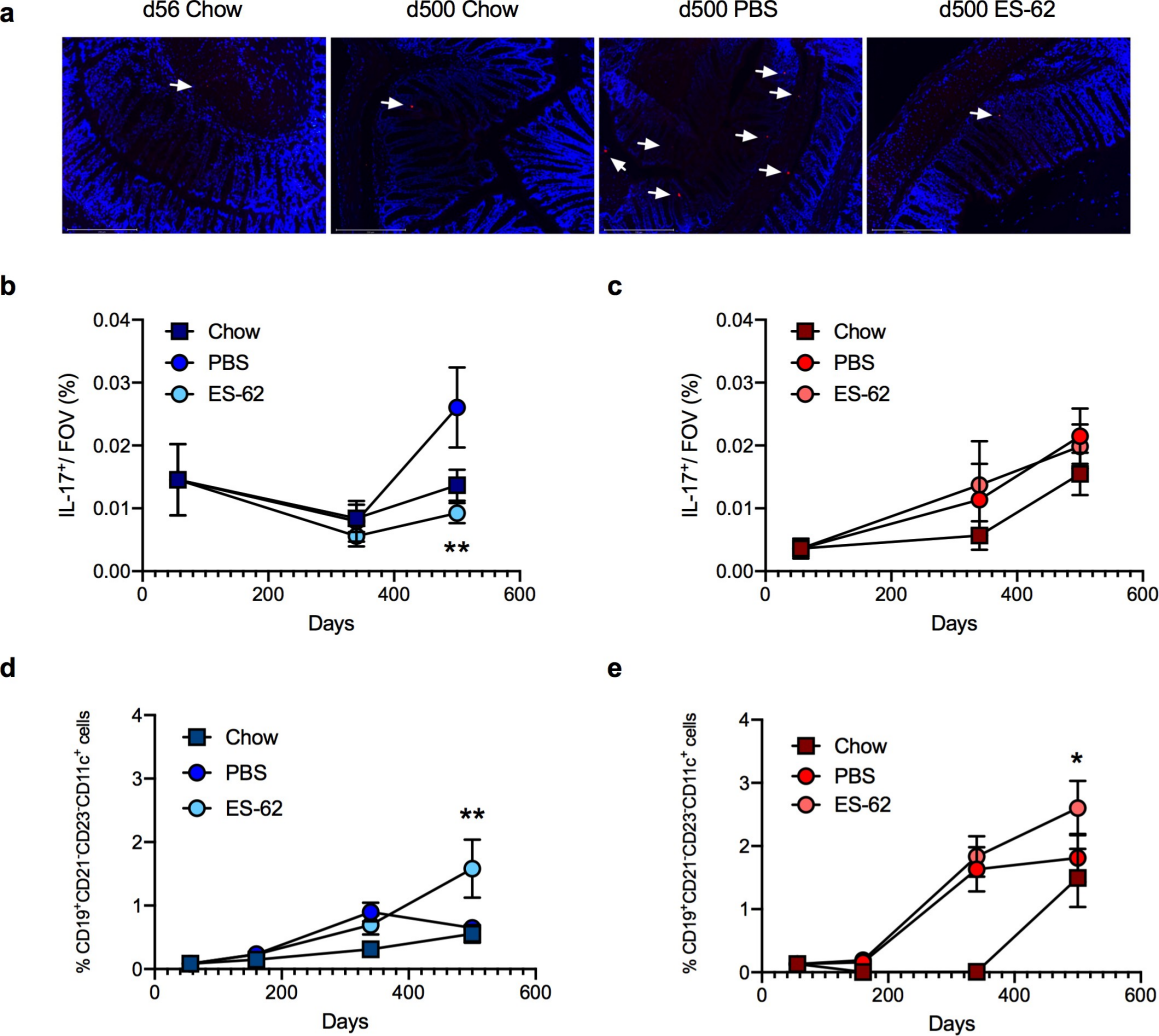
ES-62 modulates colon IL-17 and ageing-associated B cell responses in HCD mice. Representative images (a; scale bar 200 μm; d500) and quantitative analysis (b, c) of colon IL-17-expression (red, indicated by arrows; DAPI counterstain, blue): data represent mean (of triplicate analyses) values ± SEM, where n = 4–6 individual mice per time point. Splenic CD19^+^CD21^-^CD23^-^CD11c^+^ B cells (% live cells) presented as mean values ± SEM where male (d) cohort sizes: chow—d56, n = 6; d160, n = 6; d340, n = 6; d500, n = 5; HCD-PBS—d160, n = 6; d340, n = 7; d500, n = 6; HCD-ES-62—d160, n = 6; d340, n = 8; d500, n = 6 and female (e) cohort sizes: chow—d56, n = 5; d160, n = 6; d340, n = 6; d500, n = 3; HCD-PBS—d160, n = 9; d340, n = 11; d500, n = 6; HCD-ES-62—d160, n = 10; d340, n = 12; d500, n = 6. Significant differences between HCD-PBS and HCD-ES-62 cohorts are shown where *p < 0.05 and **p < 0.01.

HCD was found to accelerate and exacerbate ileum and colon pathology occurring during ageing of chow-fed mice not only in terms of an overall pathology score (scoring based on degree of cellular infiltration, epithelial erosion and villus atrophy [41]) but also by quantitative analysis of villus length/crypt morphology, basal lamina thickness, goblet cell loss and collagen deposition in both male and female mice (Fig 5A–5L; S10A–S10J Fig). ES-62 afforded significant protection against certain of these features of HCD-induced gut pathology in male, but not female, mice, specifically slowing the age-associated shortening in ileum villus length and preventing the changes in crypt morphology (ratio of crypt depth to intercrypt width [41,42]) and overall pathology in the colon (Fig 5A–5L; S10A–S10J Fig). Furthermore, reflecting the cellular infiltration and inflammation identified in the pathology scoring, whilst the male mice in the HCD-PBS cohort exhibited increased levels of IL-17 expression in colon relative to their chow-fed counterparts, this was abrogated by exposure to ES-62. By contrast, colonic IL-17 expression progressively increased with ageing in female mice, irrespective of diet or exposure to ES-62 (Fig 6A, 6B and 6C).

Age-associated B cells (CD19^+^CD21^-^CD23^-^CD11c^+^ B cells) have been proposed to promote TH1/TH17-mediated inflammation (and autoimmunity) during ageing, although they have also been reported to play roles in protective immunity, particularly with respect to viral infection [43,44]. Accumulation of these circulating cells is increased in the spleens of ageing mice, particularly as reported previously in the female cohorts [43,44] and their accumulation is accelerated by HCD (Fig 6D and 6E). Perhaps reflecting their reported relatively high phosphorylcholine (PC)-reactivity, they are further increased in both the male and female ES-62-treated d500 HCD-treated cohorts, presumably as a consequence of chronic exposure to the PC-containing worm-derived molecule [4,5].

**Fig 7:**
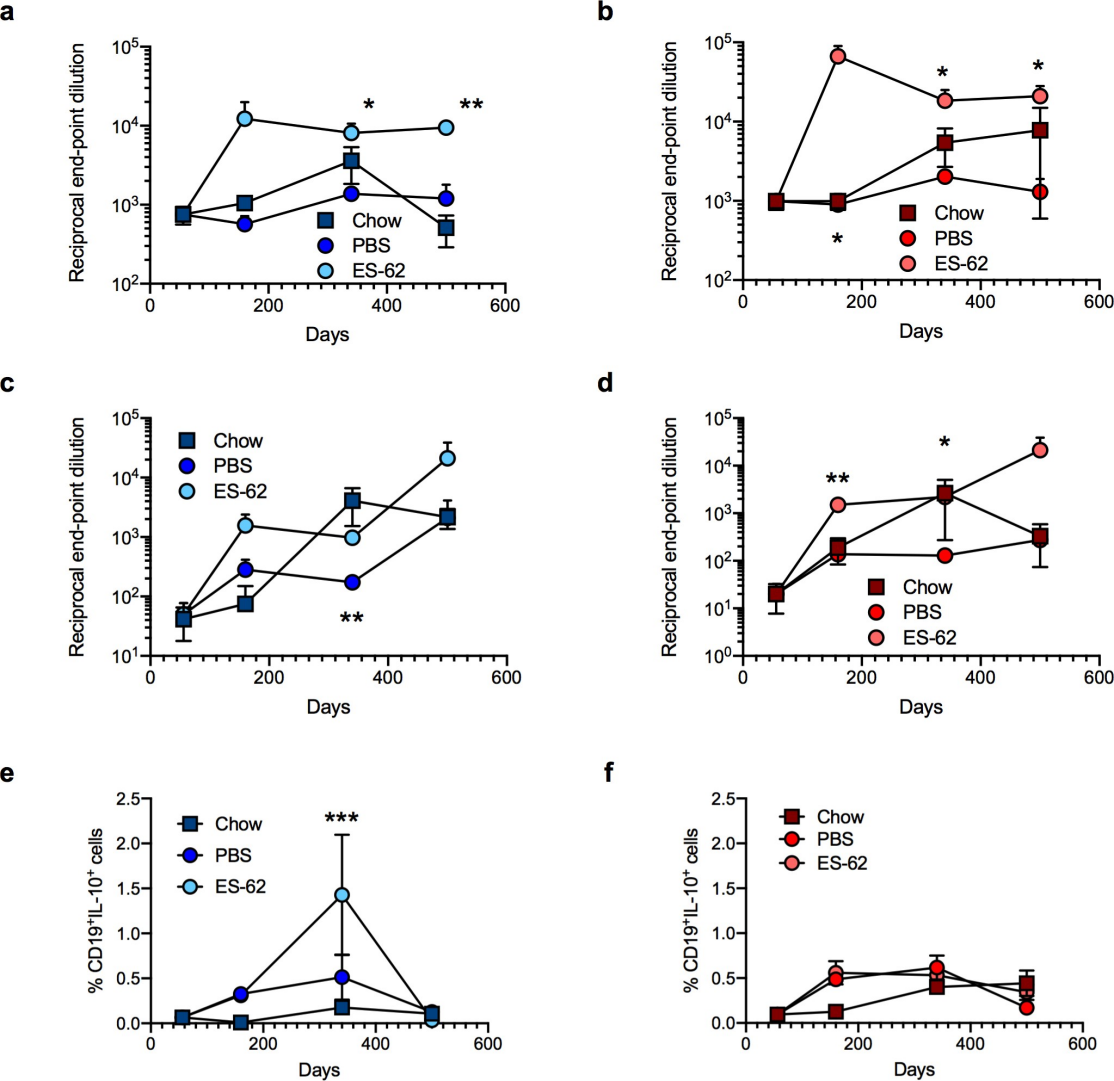
ES-62 modulates effector and regulatory B cell responses in HCD mice. Reciprocal end-point dilutions of anti-PC IgM (a, b) and IgG (c, d) antibodies as mean values ± SEM where male (a, c) cohort sizes: chow—d56, n = 6; d160, n = 6; d340, n = 6; d500, n = 5; HCD-PBS—d160, n = 6; d340, n = 10 (IgM), 11(IgG); d500, n = 6; HCD-ES-62—d160, n = 9; d340, n = 11; d500, n = 6 and female (b, d) cohort sizes: chow—d56, n = 5; d160, n = 5 (IgM), 6 (IgG); d340, n = 5 (IgM), 6 (IgG); d500, n = 5 (IgM), 3 (IgG); HCD-PBS—d160, n = 7 (IgM), 8 (IgG); d340, n = 8 (IgM), 7 (IgG); d500, n = 6; HCD-ES-62—d160, n = 8; d340, n = 10 (IgM), 11 (IgG); d500, n = 6. (e, f) MLN Bregs (CD19^+^IL-10^+^; % live cells) are presented as mean values ± SEM where male (e) cohort sizes: chow—d56, n = 6; d160, n = 6; d340, n = 6; d500, n = 5; HCD-PBS—d160, n = 10; d340, n = 7; d500, n = 6; HCD-ES-62—d160, n = 10; d340, n = 8; d500, n = 6 and female (f) cohort sizes: chow—d56, n = 6; d160, n = 6; d340, n = 6; d500, n = 4; HCD-PBS—d160, n = 9; d340, n = 10; d500, n = 5; HCD-ES-62—d160, n = 9; d340, n = 10; d500, n = 6. Significant differences between HCD-PBS and HCD-ES-62 cohorts are shown where *p < 0.05, **p < 0.01 and ***p < 0.001.

ES-62 enhancement of the anti-PC antibody repertoire (Fig 7A–7D) could possibly serendipitously boost health- and lifespan by helping to protect against bacterial infection in old age[45] given the increased virulence, morbidity and mortality of (PC-containing) *Streptococcus pneumoniae* and *Haemophilus influenza* in the elderly [46,47]. Interestingly, therefore, we have found HCD to reduce the levels of anti-PC antibodies in male and female mice, with this reaching statistical significance with respect to the levels of anti-PC IgG antibodies in male mice relative to the chow-fed counterparts at d340 (*p<0.05). Moreover, CD19^+^IL-10^+^Bregs can act to resolve pathogenic inflammation [48] including that associated with perturbation of the gut microbiota [49] and obesity [50] and these were found to be significantly increased in the MLN of male, but not female, d340 mice exposed to ES-62, a cohort that exhibited protection against HCD-induced gut pathology (Fig 7E and 7F).

### ES-62 acts to normalise the gut microbiota in ageing male HCD-fed mice

**Fig 8:**
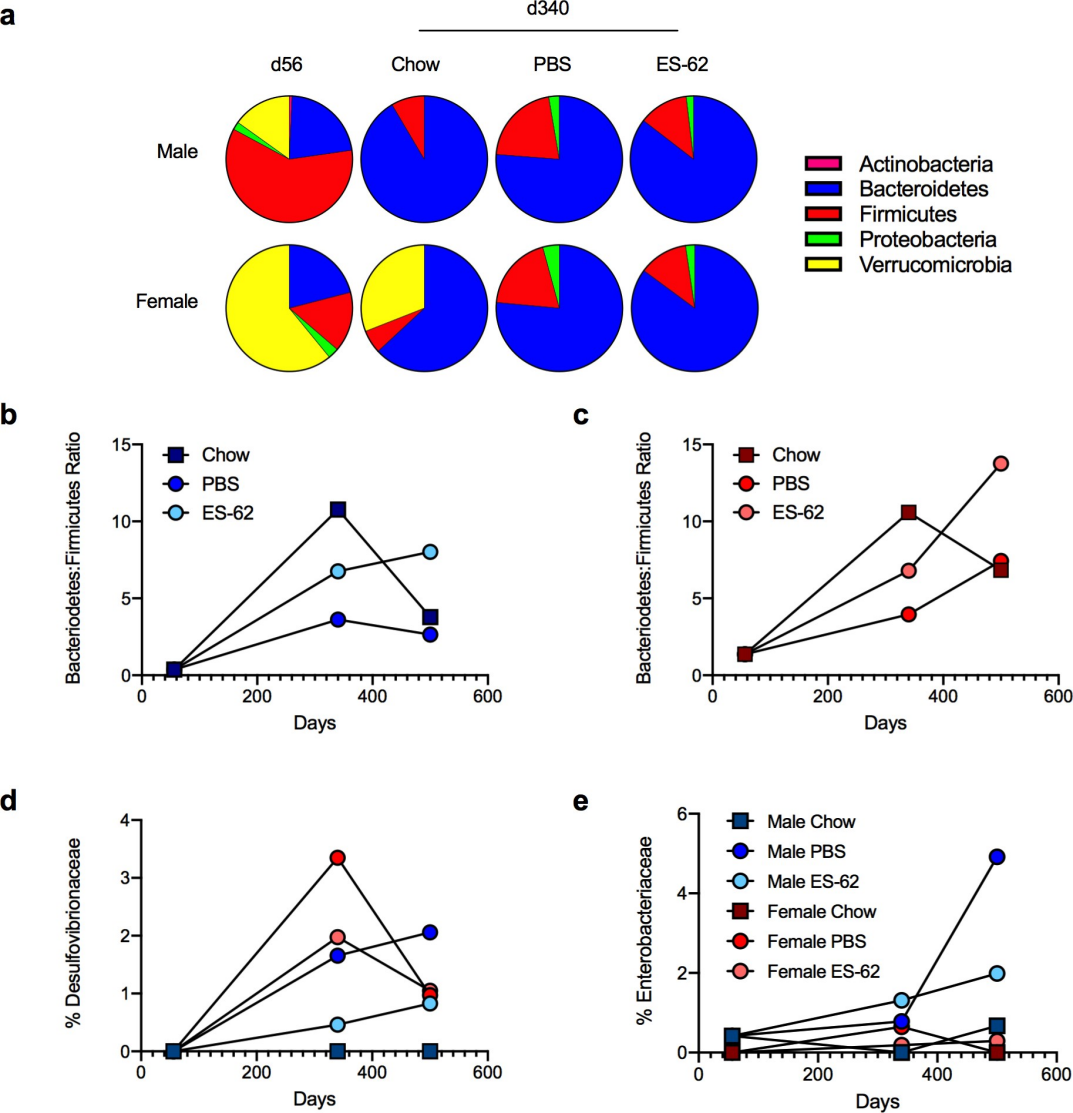
ES-62 acts to normalise the gut microbiome in HCD-fed mice. The composition of bacterial phyla in gut (ileum plus colon) faecal matter (a) of male and female chow- (d56 and d340) and HCD- (PBS- or ES-62-treated; d340) mice presenting proportion values as pie charts using pooled samples to represent each cohort. The Bacteroidetes:Firmicutes ratio (b, c) and levels of Desulfovibrionaceae (d) and Enterobacteriaceae (e) present in gut faecal (ileum plus colon) matter of male (b, d, e) and female (c, d, e) chow- and HCD- (PBS or ES-62-treated) mice (each determined as proportion values) using pooled samples to represent each cohort at the indicated time-points are shown.

To investigate whether the ES-62-mediated protection against gut inflammation and (consequent) pathology in male HCD-mice was associated with stabilization of the microbiota, we performed metagenomic analysis of faecal (ileum plus colon) material, with samples from individual mice pooled to generate a representative cohort phenotype. We found profound differences at the phylum level in the microbiota not only amongst young and ageing male and female chow-fed mice, but also to a lesser extent between aged-matched chow- and HCD-PBS mice. ES-62 acted to normalise the impact of HCD back towards the chow profile, particularly in male mice. Although it could not rescue the striking loss of Verrucomicrobia observed in either sex, ES-62 had less effect in female animals in part because young female mice exhibited much greater levels of Verrucomicrobia, proportions of which were still maintained in the chow-fed cohorts but not those undergoing HCD-accelerated ageing at d340 (Fig 8A).

Typically, whilst the ratio of Bacteriodetes:Firmicutes increases from birth through to adulthood, it subsequently remains relatively stable in healthy individuals before decreasing in old age [51]. However, obese adult humans show a decreased Bacteroidetes:Firmicutes ratio relative to aged-matched lean individuals [40]. Reflecting this, whilst the proportions of Bacteriodetes:Firmicutes in the faecal (ileum plus colon) matter of young (d56) lean male and female mice are low, mid-life adult (day 340) chow-fed mice exhibit a much higher Bacteriodetes:Firmicutes ratio which is similarly, substantially reduced in both sexes of HCD-PBS mice (Fig 8B and 8C). As expected, the Bacteroidetes:Firmicutes ratio in chow-fed mice of both sexes declined during ageing but this was more pronounced in the male relative to the female d500 chow cohorts (by ~65% and ~35%, respectively). Exposure to ES-62 protects against the HCD-induced decreases in the Bacteroidetes:Firmicutes ratio in both male and female mice and indeed, maintains, or even increases, their proportional abundance in the d500 HCD-fed cohorts (male, ~75%; female, ~130%, relative to the mid-life adult d340 chow-fed animals; Fig 8B and 8C). Perhaps critically, given the proposed roles for Proteobacteria in promoting ageing [52,53], we find the HCD-induced increases in Desulfobrionaceae (δ-Proteobacteria negatively associated with longevity [52]) to be inhibited by exposure to ES-62 although in the case of female HCD mice, this reduction was only generally to around the level found in male HCD-PBS mice at d340 (Fig 8D). Moreover, HCD induces a strong enrichment of Enterobacteriaceae (γ-Proteobacteria associated with ageing-induced fraility in *Drosophila* [53]), in the d500 cohort of male but not female mice and this outgrowth is dramatically reduced by exposure to ES-62 (Fig 8E).

### Mathematical modelling of ES-62 healthspan “signatures” in male and female HCD-fed mice

**Fig 9:**
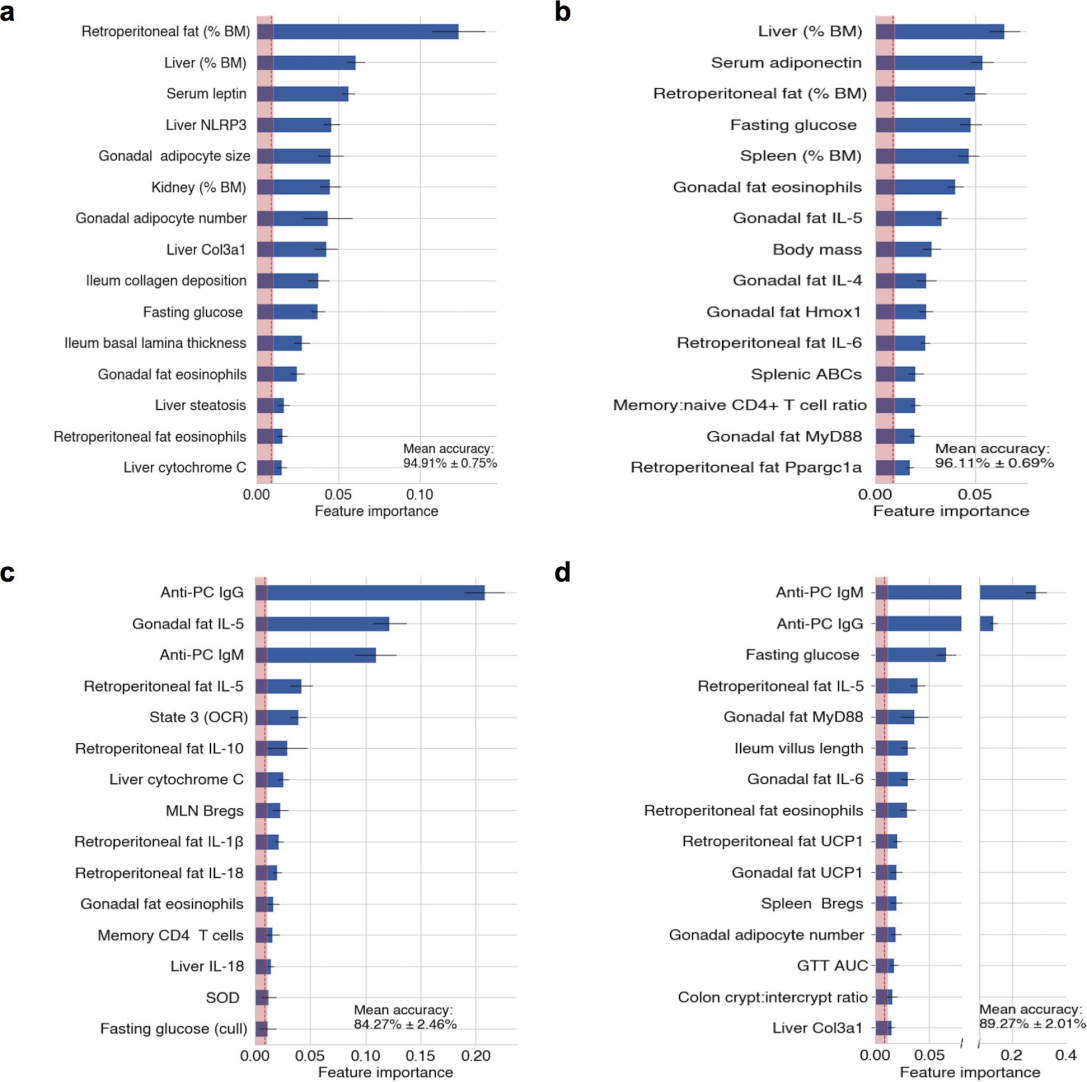
Mathematical modelling of ES-62 healthspan targets. As described in Methods, to identify the pathophysiological, metabolic and immunological variables most robustly associated with ES-62 treatment, sex, and diet, each of the associated mouse cohorts were treated as classes in supervised machine learning classification. The top predictors of (a) HCD-feeding (average 94.91% accuracy of classification), (b) sex-biased effects of HCD-feeding (average 96.11% accuracy of classification) and features most robustly indicative of ES-62-treatment of (c) female (average 84.27% accuracy of classification) and (d) male (average 89.27% accuracy of classification) HCD-fed mice are shown. Each bar represents the mean F score of the corresponding feature, horizontal black lines represent standard errors of the mean, the vertical dotted red line is the global mean F score of all features, and the shaded area is ≤ 1 standard error of the global F score mean. Features considered robustly associated with treatment are those that exceed the shaded area.

Consistent with the sexual dimorphism in the decline of tissue homeostasis resulting in metabolic and immune response dysfunction during ageing [54–56], our survival and cross-sectional intervention studies have highlighted that ES-62 differentially targets distinct pathophysiological responses during HCD-accelerated ageing in male and female mice. To distinguish the key protective healthspan signatures in each sex and to address identifying the factors associated with the ability of ES-62 to extend median lifespan in male HCD-fed mice, we have subjected our multidimensional data sets (120 pathophysiological, immunological and metabolic variables assayed on individual mice in this study) to unsupervised mathematical modelling and statistical analysis. Firstly, we validated our data set analysis by identifying the features most predictive of HCD-fed mice (Fig 9A) and as expected, this revealed (with ~95% accuracy) increases in adiposity and liver damage as well as adipokine responses associated with IR. Examination of the sex differences highlighted the inflammatory bias (Fig 9B) of the female response to HCD, demonstrated in the experimental studies, above. Reflecting this bias, prediction of the healthspan features most robustly associated with ES-62-treatment of female HCD-fed mice (Fig 9C) revealed modulation of a panel of almost exclusively, inflammatory markers (anti-PC antibodies, visceral fat eosinophils and type-1 [IL-1β, IL-18] and type-2, [IL-5, IL-10] cytokines). By contrast, whilst ES-62 treatment of male HCD-fed mice (Fig 9D) was also associated with modulation of inflammatory markers, it additionally targeted signatures associated with gut integrity (ileum villus length, colon crypt: intercrypt ratio) and metabolic tissue function (fasting glucose, gonadal adipocyte hypertrophy, visceral fat UCP1).

## Discussion

Our cross-sectional and longitudinal analysis of the impact of ES-62 on HCD-accelerated ageing has provided unparalleled depth of understanding of how a parasitic worm product functions to modulate host (patho)physiology across the lifecourse of both male and female mice. The ageing process is multifactorial, integrating the progressive loss of tissue repair and regeneration resulting from changes in the epigenetic landscape and genomic instability, deregulation of metabolic pathways and mitochondrial function, cellular senescence, stem cell exhaustion and inflamm-ageing, all of which can be impacted by (interacting) environmental factors like diet, smoking and the microbiota, as well as being subject to sexual dimorphism [23]. Perhaps not surprisingly given this complexity, our hypothesis that ES-62 might counter HCD-accelerated ageing by virtue of its potential to suppress the chronic MyD88-driven inflammation promoting metabolic deregulation, obesity and ageing, *per se* proved to be rather simplistic. Indeed, mathematical modelling of our multidimensional pathophysiological and immunometabolic data sets revealed distinct and dynamic sex- and diet-specific signatures of improved healthspan, identifying multiple targets exhibiting sexual dimorphism in terms of ES-62 responsiveness. ES-62 substantially extended the median lifespan of male HCD mice but had no effect on the longevity of their female counterparts. This male bias resulted despite its anti-inflammatory actions (e.g. promotion of type-2 responses in adipose tissue) being most strongly exhibited against female inflamm-ageing responses, and dictated that ES-62-protection against HCD-accelerated ageing could not simply be explained on the basis of its ability to suppress and resolve chronic inflammation. Rather, our signatures underlined that ES-62 additionally preferentially acted to reduce dysbiosis of the gut microbiota and protect against gut inflammation and pathology and deregulation of adipose tissue homeostasis in male HCD-fed mice. However, not all markers of gut health were improved following treatment with ES-62, for example, we could find no evidence for changes in mucin or goblet cell levels. Nevertheless, overall our data support improved homeostasis of the gut-metabolic tissue axis as contributing to ES-62 extension of lifespan in obese male mice.

To further understand the protective effects of ES-62 on the gut-metabolic tissue axis, it is worth considering that the actions identified by machine learning to be most robustly predictive of ES-62 improvement of healthspan in either sex are actually anti-PC IgM and IgG antibody responses. That female mice exhibit comparable levels of these antibodies may argue against a role for them in promoting extension of lifespan in male HCD-fed mice: however, the well-documented evidence that females generally make stronger immune responses and consequently are better at fighting infection *per se* [56–58], raises the possibility that ES-62 induction of such “natural” antibodies may be particularly important to its lifespan-extending effects in male mice. Pertinently, male mice make poor ABC responses and have their anti-PC IgG responses significantly reduced by HCD, perhaps making them innately more susceptible to atherosclerosis [59] and (PC-containing) bacterial infection in old age [45–47]. Although ES-62-mediated protection against lupus-accelerated atherosclerosis being accompanied by the induction of anti-PC antibodies [60] may resonate with this hypothesis, these antibodies do not constitute the T15 idiotype [60] generally associated with protection against atherosclerosis [59] and pneumococcal infection [45–47]. However, other idiotypes may exhibit protective properties, although a recent report on an anti-PC monoclonal antibody with a specificity distinct from the T15 idiotype revealed that a Western style diet, which resulted in severe hyperlipidemia, could overcome the athero-protective effects of this antibody [61]. Intriguingly, it has recently been reported that “natural” antibodies can convert effector B cells and DCs to particular regulatory phenotypes, characterized by their low CD1d expression, that can protect against pathogenic kidney inflammation by suppressing IFNγ, but yet maintain or even increase IL-4 production by NKT cells [62,63]. Modulation of the NKT response is associated with reduced CD1d-lipid presentation in conjunction with PD-1/PDL-1 and/or IL-10 interactions, but no FoxP3 Treg induction is required [62,63]. Perhaps pertinently therefore, by differentially sensing dietary and endogeneous lipids presented via CD1d on adipocytes, NKT cells have been proposed to play both pathogenic (promoting adipose tissue type-1 inflammation and insulin resistance) and also protective (not only via IL-4, IL-5 and IL-10 production, but also by induction of beige cells and thermogenesis and “sensing” of microbiome dysbiosis) roles in obesity [64–68]. By rebalancing NKT responses towards those promoting healthy, lean adipose tissue [64–67,69], CD1d^+^ Breg induction could explain some of the protective effects of ES-62. In addition to NKT cells, ILC2 cells have been reported to be major sources of IL-5 in adipose tissue that promote type-2 responses to regulate obesity in mice [25,68]: we therefore plan to attempt to determine whether they are also ES-62 targets, although we have not been able to detect any significant effects on their very low levels (~0.1% live SVF cells) in the visceral adipose tissues examined to date. Intriguingly, there is increasing evidence that NKT cells, like other cells of the innate immune system including eosinophils and macrophages, exhibit (infection and disease-dependent) sexual dimorphisms in their responses: since these sex-biases are influenced by each of sex chromosomes, hormones and bidirectional interactions with the microbiota, they may vary over the lifecourse impacting on disease susceptibility and longevity [20,57,58,70]. In any case, since we cannot correlate the ES-62 promotion of a type-2 inflammatory adipose microenvironment with protection against adipocyte hypertrophy (that normally results in increased adipokine secretion, hypoxia and adipocyte cell death causing inflammation and fibrosis) in either the gonadal or retroperitoneal visceral fat beds in HCD-fed mice, we consider that an ILC2/eosinophil/M2-like macrophage-driven immunoregulatory mechanism is unlikely to contribute to the helminth product’s extension of median lifespan of male HCD-fed mice.

Intriguingly, given that we have recently shown ES-62 to “fine-tune”the gut microbiota in the absence of inflammation in healthy male DBA/1 mice [12], there is increasing evidence that NKT cells can also regulate the gut microbiota and gut health (and vice versa) by sensing bacterial, dietary and endogeneous lipids [71–74]. Thus, HCD-induced imbalance of the lipids sensed, could generate a NKT-dependent pathogenic cycle leading to gut inflammation and loss of barrier integrity and defective energy consumption and fat storage, which is perpetuated by the ongoing dysbiosis in obesity [64–67,71–74]. Supporting this hypothesis, CD1d-deficient mice exhibit both enrichment of segmented filamentous bacteria (and consequently increased Th17 responses) and depletion of *Akkermansia*, a genus in the phylum, Verrucomicrobia, dramatically lost in ageing male and particularly, (HCD-accelerated) female mice. Moreover, and somewhat reminiscent of what we see with ES-62, mice deficient in CD1d, specifically in their CD11c^+^ cells, exhibit disruption of intestinal homeostasis whilst exhibiting no change in gross morphology or levels of mucin and goblet cells [74]. Thus, the ability of ES-62 to induce anti-PC “natural” antibodies that could, by induction of CD1d^low^ regulatory DC and Bregs, impact on NKT sensing and regulation of the gut microbiota may provide an important “sensor” mechanism in its ability to maintain immunometabolic homeostasis in HCD-accelerated ageing. Certainly, the protection afforded by ES-62 against collagen-induced arthritis and associated pathogenic TH17-mediated inflammation is associated with restoration of the defective Breg/effector B cell balance, normalisation of the gut microbiome and stabilisation of gut barrier integrity in this model of rheumatoid arthritis in male mice [12]. Clearly therefore, these ideas could be worthy of future exploration in order to more fully determine whether there is an overarching mechanism by which ES-62 acts to improve healthspan and lifespan in the HCD model of obesity-accelerated ageing.

In the meantime, further support for our hypothesis that ES-62-mediated extension of lifespan relates to improved gut health is provided by emerging evidence from *Drosophila* research, which suggests that lifespan is limited by gut pathology such that intestinal epithelial barrier integrity appears to be a more effective predictor of mortality than chronological age [75,76]. Indeed, it has been proposed that the extension of lifespan in response to dietary restriction in female flies may reflect the effectiveness of this regimen in reducing the profound gut pathology observed during ageing [77]. Whilst generally stable in healthy adults, the gut microbiome can undergo dynamic changes, particularly in early and late life and during inflammatory and metabolic disorders [78], that contribute to loss of intestinal barrier integrity [37,38,53] and impact directly on obesity and longevity [39,40]. For example, the Verrucomicrobia species that we found to be profoundly depleted during (HCD-accelerated) ageing have been reported to be a marker of gut health. This reflects primarily the ability of Akkermansia species, like *Akkermansia muciniphilia* to exploit the outer mucus layer as a food-source and, by degrading complex mucin glycoproteins to produce acetate and propionate, support growth of butyrate-producing species to provide an energy source for the gut epithelium cells to renew and promote barrier integrity. Indeed, treatment with *A*. *muciniphilia* has been shown to restore mucus layer thickness and prevent metabolic disorders in mice fed a high fat diet (*reviewed* [79]). In addition, and contributing to their perception as “healthy” bacteria, Verrucomicrobia have been proposed to foster Treg generation and insulin sensitivity [79,80].

Our observed sexual dimorphism in gut Verrucomicrobia levels and their depletion during ageing of obese mice may suggest that, as with fruit flies [77], female relative to male C57BL/6J mice may be particularly vulnerable to (HCD-induced) gut pathology during ageing. The failure of ES-62 to restore these commensals, and promote Treg responses in general [4], may go some way to explaining why the helminth product does not extend lifespan in female HCD-fed mice but only in the male cohorts, which given their lower levels of Verrucomicrobia may be innately less susceptible to the pathological consequences of gut dysfunction. By contrast, a microbiome-protective sexual dimorphism was revealed in the actions of ES-62 against pathogenic Proteobacteria, enrichment of which promotes loss of gut barrier integrity. Thus, although the HCD- and ageing-associated enrichment of the Desulfovibrionaceae family of δ-Proteobacteria was countered by exposure to ES-62 in both sexes, rescue was more pronounced in male mice. More strikingly, whilst HCD induces a strong enrichment of Enterobacteriaceae in ageing male but not female mice, this outgrowth is essentially ablated by ES-62. Consistent with this sexual dimorphism contributing to the ability of ES-62 to extend median lifespan in male HCD-fed mice, whilst enrichment of Enterobacteriaceae positively correlates with ageing-induced frailty in flies [53], the Desulfovibrionaceae negatively correlate with longevity and have previously been shown to be reduced following dietary restriction in male C57BL/6J mice [52].

## Conclusions

In this unique study, we have investigated the effect of a defined parasitic worm product, ES-62, on obesity-accelerated ageing essentially throughout the entire lifespan of mice. It is perhaps remarkable that, when administered only weekly and at low doses, ES-62 (differentially) improves pathophysiological, immunological and metabolic parameters of healthspan in male and female HCD-fed mice and substantially increases median lifespan of the male animals. Indeed, an important outcome of the study has been the revelation of how strongly each of diet and sex can drive, via distinct mechanisms, the deregulation of immunometabolic networks to impact on the ageing process. Thus, our machine learning generated signatures predictive of the protective sex-specific actions of ES-62 may go some way to explaining the often observed sexual dimorphism associated with interventions that impact on health- and lifespan and hence, emphasise the need for well-defined ageing markers that identify and stratify population cohorts for effective therapeutic responsiveness. Perhaps most surprisingly, our data suggest that the lifespan-promoting effects of ES-62 cannot (solely) be explained by its anti-inflammatory actions. Rather, ES-62 appears to preferentially target the gut-metabolic tissue axis in male HCD-mice, acting to normalise gut microbiome changes and loss of intestinal barrier integrity to protect against mortality otherwise resulting from the consequent dysregulation of immunometabolic pathways during obesity-accelerated ageing. The precise mechanism by which ES-62 achieves this remains to be definitively established, but we now plan to focus on defining whether the worm-derived product maintains gut microbiome homeostasis by acting directly as a quorum sensor or by impacting on innate (anti-PC/CD1d [Breg, DC, intestinal epithelial cell]/NKT-mediated) regulation of commensals to promote gut health and homeostatically (re)program immune responses.

## Methods

### Ethics statement

All protocols and procedures were approved by The University of Glasgow Animal Welfare and Ethical Review Board and undertaken in accordance with a UK Home Office Project Licence (60/4504), following the “principles of laboratory animal care” (NIH Publication No. 86–23, revised 1985).

### Mouse husbandry: Longevity and cross-sectional healthspan studies

Male and female C57BL/6J mice (Envigo, UK) were housed in the Central Research Facility (University of Glasgow, Scotland) and maintained at 22°C under a 12 h light/dark cycle. Lifespan (survival study) cohort mice (all groups n = 24) were housed in same sex groups of 2 to 4 animals under specific pathogen-free conditions within individually ventilated cages (Techniplast, UK). Mice had *ad libitum* access to water and normal chow (CRM-P; SDS, UK; Oil, 3.36%; Protein 18.35%; Fibre, 4.23%: Sugar 3.9%; Atwater fuel energy from Oil, 9.08%; Protein, 22.03%: Carbohydrate, 68.9%) plus 150 ppm Fenbendazole, until 10 weeks of age when the diet was switched to Western Diet RD, here denoted high calorie diet (HCD; Fat, 21.4%; Protein, 17.5; Fibre, 3.5%; Sucrose 33%; Atwater fuel energy from Fat, 42%; Protein, 15%: Carbohydrate, 43%) plus 150 ppm Fenbendazole. Mice were administered PBS or purified ES-62 (1 μg) weekly via the subcutaneous route from 9 weeks old. Endotoxin-free ES-62 was purified from spent culture medium as described in detail previously [81]. Individuals in the lifespan study were monitored daily, weighed twice-weekly and analysed for grip strength monthly, but otherwise left undisturbed until they died. A pre-weighed amount of food was administered via wire bar lid food hopper. The average consumption/mouse/day was calculated at the end of a 72-hour monitoring period monthly. Grip strength monitoring was carried out as per manufacturer’s guidelines (Ugo Basile®, Italy) using a Gripometer, measuring the peak grip force (GF) of the front limbs. The mouse was lowered towards a T-shaped grasp bar, once the animal gripped the bar it was gently pulled away and the peak amplifier recorded the amount of resistance. An average of three measurements was described as the mean GF.

Survival was assessed from 48 female (HCD-PBS, n = 24; HCD-ES-62, n = 24) and 48 male (HCD-PBS, n = 24; HCD-ES-62, n = 24) mice, with all animals dead by the time of this report. Kaplan-Meier survival curves were constructed using known birth and death dates, with differences between groups evaluated using the log-rank test. If death appeared imminent (as assessed by our humane end-points) mice were weighed, euthanized and examined for macroscopic pathological changes using previously described protocols [82,83] with this date considered date of death.

Additional cross-sectional healthspan cohorts of mice were sacrificed at 56 (Chow, n = 6), 160 (HCD, n = 10/group; Chow, n = 6), 340 (HCD, n = 12/group; Chow, n = 6) and 500 (HCD, n = 6/group; Chow, n = 5) days of age and tissues were harvested for processing. At sacrifice, blood was collected and the serum isolated and stored at -20°C in endotoxin-free Eppendorf tubes. Liver was immediately removed post-mortem and mitochondria prepared for Seahorse analysis [84]. Blood, spleen, mesenteric lymph nodes (MLN) and visceral (gonadal: epididymal and parametrial fat pads, and retroperitoneal: dorsal fat pad directly behind the kidneys and attached to the peritoneum proposed as relevant models for human intra-abdominal adipose tissue [85,86]) fat tissue were prepared for flow cytometry analysis. Ileum and colon faecal contents and liver and visceral adipose tissues were collected in sterile endotoxin-free Eppendorf tubes, snap frozen and stored at -80°C. In addition, all tissue samples were fixed in 10% neutral buffered formalin and embedded in either paraffin or flash frozen in OCT and stored at -80°C for histological analysis.

### Tissue processing, histology, and immunofluorescence

Paraffin embedded tissues were sectioned at 5–6 μm and OCT tissues were cryosectioned (Thermoscientific, UK) at 8–10 μm thickness between -14 to -30°C. All tissues were haematoxylin and eosin (H&E) stained, gut and liver tissue sections were also stained with Gömöri’s Trichrome using previously described methods [12]. Prior to immunofluorescence (IF) staining, antigen retrieval (10 mM citrate buffer, pH 6) was performed and the sections were avidin/biotin blocked (Vectorlabs, UK). Briefly, pancreatic insulin and glucagon were stained with rabbit anti-insulin (1/200 dilution: Catalogue number Ab181547 Abcam, UK) and mouse anti-glucagon antibodies (1/200 dilution: Catalogue number Ab10988 Abcam, UK) overnight, followed by goat anti-rabbit IgG Alexafluor 488 (1/400 dilution: Abcam, UK) and goat anti-mouse IgG Alexafluor 647 (1/400 dilution: Catalogue A28181 Invitrogen, UK). IL-17 was detected using goat anti-mouse IL-17 (1/100 dilution: Catalogue number Af-421-na R&D Systems, UK) followed by biotinylated rabbit anti-goat IgG (1/200 dilution: Catalogue number 31732 Invitrogen, UK) with streptavidin Alexafluor 647 (1/200: dilution Catalogue number S21374 Invitrogen, UK). IF sections were mounted with Vectashield with DAPI (Vectorlabs, UK) and images were acquired with an EVOS FL Auto 2 system (Thermofisher, UK).

### Image quantification and analysis

Images were quantified by Volocity (PerkinElmer, USA) or ImageJ Software. The total pancreatic islet number and area (pixels) were quantified and total IF-labelled glucagon and insulin fluorescence (pixels) identified and thresholded across the entire field of view (FOV) using set intensity parameters. Liver fat droplet deposition was calculated as a percentage of thresholded “white area” relative to the total area of liver tissue in FOV. Adipose cell nuclei were identified, thresholded and counted using set intensity parameters and the particle analyser plugin. Adipocount software (CSBIO) was used to quantify the number and area of individual adipocytes in the FOV. In ileum and colon tissue basal lamina width, villi length (from crypt to tip) or crypt depth was measured in three separate areas in the FOV and averaged. Collagen deposition was quantified by identifying percentage green/blue staining in FOV using the ColourDeconvolution plugin. A gut tissue pathology scoring protocol was developed from the methods described by Erben *et al*. [41]. Images were given an unbiased score for each described parameter such as goblet cell numbers. The overall pathology score was calculated (average of degree of cellular infiltration, epithelial erosion and villus/crypt atrophy).

### Glucose tolerance testing and HOMA-IR

Mice were fasted overnight prior to testing. Throughout the procedure blood samples were obtained via caudal vein venesection and measured using an accu-chek performa (Roche, UK) or OneTouch Ultra (Lifescan, UK) glucometer. A fasting glucose (mmol/L) measurement was taken prior to the intraperitoneal administration of 20% glucose solution (dosage, 2g glucose/kg body mass) and subsequent glucose tolerance measurements were recorded at 15, 30, 60 and 120 minutes post injection, with the glucose tolerance expressed as the area under the curve (AUC) over this 120 minute period [87]. Serum insulin, leptin and adiponectin were assessed using the mouse metabolic and adiponectin MSD kits as per manufacturer’s guidelines (Meso Scale Diagnostics, USA). Insulin resistance was estimated using the Homeostasis Model Assessment for Insulin Resistance (HOMA-IR) index using the formula described previously [88], HOMA-IR index = [fasting glucose (mmol/L) x fasting insulin (mIU/L)]/22.5).

### Flow cytometry

Cells from spleen, MLN, whole blood and the stromal vascular fraction (SVF) of gonadal and retroperitoneal adipose tissues were suspended in FACS buffer (2.5% BSA; 0.5 mM EDTA, in PBS) following treatment with red blood cell-lysis buffer (eBioscience, UK). Adipose tissue was digested with 1 mg/ml collagenase type II (Sigma, UK) and passed through 100 μM cell filters to generate a single cell suspension. Cells were washed in FACS buffer and incubated with Fc block (Biolegend, UK) before staining with the relevant antibodies/streptavidin conjugates, all of which were purchased from BioLegend, UK unless indicated otherwise. For eosinophils, cells were analysed for SiglecF (PE: Catalogue number 552126; BD Bioscience, UK) expression and for macrophage phenotypes, F4/80 (Biotin: Catalogue number 123105 with SA-ef780), CD301b (APC: Catalogue number 146813) or CD206 (APC: Catalogue number 141707) and CD11c (PeCy7: Catalogue number 117318) expression was determined. Whole blood was red-cell lysed, washed in FACS buffer before incubation with Fc block and the relevant antibodies. T lymphocytes were incubated with antibodies specific for mouse CD3 (FITC or PE: Catalogue number 100203 or 100205), CD4 (PE: Catalogue number 100407 or APC-ef780: Catalogue number 47-0042-82 eBioscience, UK), CD8 (PeCy7: Catalogue number 25-0081-82, eBioscience, UK), CD45RB (FITC: Catalogue number 103305 or APC-ef780: Catalogue number 47-0451-82, Invitrogen, UK) and CD44 (PerCP: Catalogue number 103036). B lymphocytes from the spleen and MLNs were labelled with antibodies specific for mouse CD19 (AF700: Catalogue number 115527), CD23 (PeCy7 or AF488: Catalogue number 101613 or 101609), CD21 (PE: Catalogue number 123409), CD11c (FITC or PeCy7: Catalogue number 117305 or 117318), IgD (PerCP/Cy5.5: Catalogue number 405709), IgM (Biotin: Catalogue number 406503; Strepavidin Bv450), IL-10 (PE: Catalogue number 505007). Fixable viability stain (APC-ef780 or and v450: Catalogue number 65-0865-14 or 65-0863-14; Invitrogen, UK) was used to select for live cells and, for analysis of IL-10^+^ regulatory B cells (Bregs), lymphocytes were stimulated with PMA, ionomycin, Brefeldin A and LPS (Sigma, UK) as described previously [12]. Data were acquired using a BD LSRII flow cytometer and populations were gated using isotype and fluorescence minus one (FMO) controls using FlowJo, LLC analysis software (Tree Star/ BD, USA) as described previously [12]. Exemplar gating strategies are presented (S11 Fig).

### Serum antibody ELISA

Anti-PC-BSA IgG and IgM antibodies in serum [81] were quantified using a reciprocal end point dilution method. Briefly, high-binding 96 well ELISA plates were coated with PC-BSA overnight at 4°C before washing and blocking with 1% BSA/PBS. Serum was initially diluted 1:100 and then serially diluted three-fold until 1:218700, incubated with HRP-conjugated goat anti-mouse IgG or IgM (1:10,000; 1:6,000 in 10% FBS/PBS) prior to developing with TMB, terminating with 2M sulphuric acid and read at an optical density of 450 nm.

### Seahorse XF assay and REDOX parameters

The XF Mitostress assay (Agilent Technologies, UK) was performed as previously described [84]. Briefly, hepatic mitochondria were isolated and added to an XF culture plate at a concentration of 10 μg per well. A pre-soaked eXF cartridge was prepared [89], the culture plate loaded into an XF24 Analyser (Agilent Technologies, UK) and the assay initiated. Oxygen consumption rate (OCR) was measured in substrate alone (10 mM pyruvate, 2 mM malate), during state 3 (ADP [4 mM]), state 4 (oligomycin [2.5 μg/ml]), state 3u (FCCP (carbonyl cyanide 4- (trifluoromethoxy) phenylhydrazone) [4 μM]), and lastly antimycin A with rotenone [both 4 μM]. All reagents were sourced from Sigma, UK. Superoxide dismutase (SOD) and protein carbonyl activities were quantified in liver tissue homogenates using Total SOD activity assay and Carbonyl assay kits, respectively in line with the manufacturer’s guidelines (Cayman Chemical Company, Estonia).

### qRT-PCR

mRNA was extracted from liver and gonadal and retroperitoneal adipose tissue using the RNeasy Lipid Tissue mini kit (Qiagen, Germany), or tissue was lysed in QIAzol prior to mRNA extraction using the DNA away RNA extraction kit (NBS Biologicals, UK). mRNA was transcribed into cDNA using the High Capacity cDNA Reverse Transcriptase kit (Applied Biosystems, Life Technology, UK) for use with Applied Biosystems Quant Studio 7 and KiCqStart® qPCR ready mix (Sigma-Aldrich) and KiCqStart^™^ Primers. Data were normalized to the housekeeping gene β-actin to obtain the ΔCT measurements that were used to calculate 2^-ΔCT values. Primer sequences were β-actin (forward—GATGTATGAAGGCTTTGGTC, reverse—TGTGCACTTTTATTGGTCTC), IL-1β (forward—GTGATATTCTCCATGAGCTTTG, reverse—TCTTCTTTGGGTATTGCTTG), IL-4 (forward–CTGGATTCATCGATA AGCTG, reverse—TTTGCATGATGCTCTTTAGG), IL-5 (forward–CCCTACTCATAAAAATCACCAG, reverse—TTGGAATAGCATTTCCACAG), IL-18 (forward–AAATGGAGACCTGGAATCAG, reverse—CCTCTTACTTCACTGTCTTTG), cytochrome C (forward—CCGGAACGAATTAAAAATGG, reverse–TCTGTGTAAGAGAATC CAGC), HMOX-1 (forward—CATGAAGAACTTTCAGAAGGG, reverse–TAGATATG GTACAAGGAAGCC) and NLRP3 (forward—GATGCTGGAATTAGACAACTG, reverse—GTACATTTCACCCAACTGTAG).

### Metagenomics

Genomic DNA was extracted from the ileum and colon faecal matter using the QIAamp DNA Stool Mini Kit (Qiagen). Colon and ileum DNA were combined and samples were pooled on a group basis for shotgun metagenomic analysis using the Ion Torrent PGM^™^ platform. Pooled DNA (100 ng) was fragmented and barcoded using the NEBNext® Fast DNA Fragmentation and Library Prep Set for Ion Torrent^™^ (NEB Inc, UK) and IonXpress Barcode Adapters kit (ThermoFisher Scientific) respectively. The quality and quantity of all barcoded DNA libraries were analysed using the High Sensitivity DNA analysis kit (Agilent, UK) on the 2100 Bioanalyzer Instrument and Qubit Fluorometer (ThermoFisher Scientific). Samples were prepared using the Ion PGM^™^ Hi-Q^™^ View OT2 and Ion PGM^™^ Hi-Q^™^ View Sequencing kits (ThermoFisher Scientific) and 4 barcoded libraries were combined per Ion 316^™^ Chip kit (ThermoFisher Scientific). Data were analysed using MG-RAST and the number of reads per phylum, class, order, family or genera of species of interest were normalized against all bacteria present. Sequencing runs can be accessed using MG-RAST IDs mgl675297, mgl675300, mgl675312, mgl675291, mgl675309, mgl675318, mgl675306, mgl675294, mgl675321, mgl675315, mgl675285, mgl675288, mgl675324, mgl675303.

### Machine learning

To identify the metabolic and inflammatory variables most robustly associated with ES-62 treatment, sex, and diet, each of those groups was treated as classes in supervised machine learning classification. Predictors, or input matrix, consisted of 120 pathophysiological, immunological, and metabolic features determined from our cross-sectional chow- and HCD-fed male and female cohorts (comprising 158 mice, 79 males and 79 females). To preserve sample sizes, missing values were imputed using the median of all the available values from other mice for that feature. Each feature was then standardised by centring it around the mean and scaling to unit variance. Models were trained using 5-fold cross-validation on a 75% split of the full dataset and tested on the remaining unseen 25%. The performance of seven common algorithms was compared (logistic regression, naive Bayes, support vector machines, k nearest neighbours, decision tree, random forests, and gradient boosted trees). We chose to further tune the gradient boosted trees models for inference. Tuning of the gradient boosted trees entailed five rounds of repeated stratified 5-fold splits of the training set in each of which there was a randomised search of parameter space for column sampling size, learning rate, minimum child weight, number of estimators, and maximum tree depth. This generated an ensemble of tuned models from which we selected the best 15 models based on their accuracy in correctly classifying mice from the test set. We then used the resulting distribution of model outputs for ranking the importance of features (Gini index) in correctly classifying the test set mice. Machine learning was performed in Scikit-Learn [90] and associated plots using Seaborn [91].

### Statistical analysis

All data were analysed using GraphPad Prism 6 or 8 software using unpaired student T-tests, one or two-way ANOVA with Fishers LSD post-test for parametric data or Kruskal-Wallis test and Dunn’s post-test for non-parametric data. For clarity, only significant differences between the HCD-PBS and HCD-ES-62 cohorts are shown on the figures, where significance is denoted by *p < 0.05, **p < 0.01 and ***p < 0.001.

## Supporting information

S1 Table Descriptive statistics of the longevity cohort. ^a^Cox regression analysis of PBS- and ES-62 -treated mice. Age at death was analysed in pooled male and female PBS and ES-62 mice using Cox regression analysis (SPSS Statistics v22). The independent variables, Treatment (PBS or ES-62) and Sex (male or female), were replaced with a set of indicator variables to denote the presence or absence of category membership. B is the un-standardised regression coefficient and its standard error is SE, its Wald test statistic value, Wald, the degrees of freedom, df and the significance, Sig. Exp(B) for the covariate of interest is the predicted change in the hazard ratio for a unit increase in the predictor. Sample size = 48 for PBS- and 48 for ES-62-treated mice. ^b^Descriptive statistics of this longevity study were derived using the GraphPad Prism8 logrank test survival software package.
(PDF)

S2 Table Pathology identified post-mortem in mice from the lifespan cohort. Individual mice (each cohort n = 24) in the lifespan study were monitored daily, weighed twice-weekly and analysed for grip strength monthly, but otherwise left undisturbed until they died (natural death). If death appeared imminent (as assessed by our humane end-points) mice were weighed, euthanised and examined for macroscopic pathological changes using previously described protocols[82,83] with this date considered date of death. The numbers of mice displaying one or more pathologies post mortem as well those undergoing natural death or euthanasia in each cohort is shown.
(PDF)

S1 Fig Correlating body mass with age. Correlation of age of death of male (a, c, e, g) and female (b, d, f, h) HCD- (PBS- and ES-62-treated) mice with (a, b) BM at d116; (c, d) BM at d160; (e,f) BM at d340; (g,h) BM at d500.
(EPS)

S2 Fig Body mass of survival and cross-sectional cohorts. Longitudinal analysis of food intake (a) and grip strength (b) measurements was undertaken where data are presented as mean values ± SEM of the individual mice surviving at the indicated time-points. Longitudinal analysis of BM measurements of the individual male (c) and female (d) HCD-fed mice in the survival (data shown in Fig 1) and indicated cross-sectional cohorts where data are presented as mean values ± SEM of the mice surviving or culled at the indicated timepoints. HCD-male and -female mice exhibited substantially higher Body Mass (BM) than their aged-matched chow control groups and male mice were significantly heavier at cull than their female counterparts (***p<0.001) at each timepoint (e & f). Cross-sectional cohorts of chow (d56, n = 6; d160, n = 6; d340, n = 6 and d500, n = 5) and PBS- or ES-62-treated HCD mice (d160, n = 10; d340, n = 12 and d500, n = 6) at cull, where data are presented as mean values ± SEM of individual mice.
(EPS)

S3 Fig ES-62 and HCD-modulation of adipocyte health in retroperitoneal fat. Representative images (scale bar 100 μm) of retroperitoneal fat from male (a) and female (c) chow- and HCD- (PBS- or ES-62-treated) mice stained with H & E and resultant quantitative analysis of adipocyte size where data are presented as the mean values ± SEM of individual male (b) and female (d) mice and the values for each mouse are derived from n = 3 replicate analyses. Levels of eosinophils in retroperitoneal fat are presented as the mean values ± SEM at each time point where male (e) cohort sizes are: chow—d56, n = 5; d160, n = 3; d340, n = 5; d500, n = 5; HCD-PBS—d160, n = 9; d340, n = 10; d500, n = 6; HCD-ES-62—d160, n = 9; d340, n = 12; d500, n = 6 and female (f) cohort sizes are: chow—d56, n = 6; d160, n = 6; d340, n = 6; d500, n = 5; HCD-PBS—d160, n = 4; d340, n = 11; d500, n = 6; HCD-ES-62—d160, n = 6; d340, n = 12; d500, n = 6. qRT-PCR analysis of IL-5 and IL-4 mRNA expression in retroperitoneal fat from chow- and HCD-fed (PBS- or ES-62-treated) mice is presented (g-j) as mean 2^ΔCT values ± SEM of individual mice and the values for each mouse are means of n = 3 replicate analyses. Male cohort sizes: chow—d56, n = 5; d160, n = 3; d340, n = 4; d500, n = 2; HCD-PBS—d160, n = 10; d340, n = 11; d500, n = 6; HCD-ES-62—d160, n = 8; d340, n = 11; d500, n = 6. Female cohort sizes: chow—d56, n = 5; d160, n = 4; d340, n = 5; d500, n = 2; HCD-PBS—d160, n = 7; d340, n = 9; d500, n = 6; HCD-ES-62—d160, n = 9; d340, n = 11; d500, n = 6. The levels of F4/80^+^CD11c^-^CD301^+^ cells (k) in gonadal fat in male and female chow- and HCD- (PBS- or ES-62-treated) mice in the d160 cohorts are shown. For clarity, only significant differences between the HCD-PBS and HCD-ES-62 cohorts are shown on the figures, where significance is denoted by *p < 0.05 and ***p < 0.001. However, in (b) the chow cohort are significantly different (p<0.05) from the HCD-PBS, but not the HCD-ES-62 mice. (l) Summary of the disconnect between adipocyte health and type-2 responses in ES-62-treated HCD mice is shown.
(TIFF)

S4 Fig ES-62 and HCD-modulation of adipocyte function. Serum levels of leptin (a, b), adiponectin (c, d) and insulin (e, f) from male (a, c. e) and female (b, d, f) chow- or HCD- (PBS- or ES-62-treated) mice at cull are shown at the indicated time-points. Data are expressed as mean values ± SEM of individual mice. Male cohort sizes: chow—d56, n-5 (leptin) n = 6 (adiponectin, insulin); d160, n = 6; d340, n = 6; d500, n = 5 (leptin, adiponectin), n = 4 (insulin); HCD-PBS—d160, n = 9; d340, n = 8 (leptin), n = 9 (adiponectin, insulin); d500, n = 3 (leptin), n = 6 (adiponectin, insulin); HCD-ES-62—d160, n = 9; d340, n = 7 (leptin), n = 9 (adiponectin, insulin); d500, n = 3 (leptin) n = 6 (adiponectin, insulin) and female cohort sizes: chow—d56, n = 6; d160 and 340, n = 5 (leptin), n = 6 (adiponectin, insulin); d500, n = 3 (leptin), n = 5 (adiponectin, insulin); HCD-PBS—d160, n = 9; d340, n = 9; d500, n = 4 (leptin) n = 6 (adiponectin, insulin); HCD-ES-62—d160, n = 9; d340, n = 9; d500, n = 4 (leptin), n = 6 (adiponectin, insulin). For clarity, only significant differences between the HCD-PBS and HCD-ES-62 cohorts are shown on the figures, where significance is denoted by ***p < 0.001.
(EPS)

S5 Fig ES-62 and HCD-modulation of pancreatic function. Representative images (scale bar 500 μm) of pancreas at d160 and d340 from male (a) and female (b) mice stained for insulin (green), glucagon (red) and counterstained with DAPI (blue). Quantitative analysis of islet size (c, d) and production of insulin (e, f) and glucagon (g, h) where data are presented as the mean values (of triplicate analyses) ± SEM of individual male (c, e, g) and female (d, f, h) mice (n = 4–6) at each time-point. For clarity, only significant differences between the HCD-PBS and HCD-ES-62 cohorts are shown on the figures, where significance is denoted by **p < 0.01.
(TIFF)

S6 Fig ES-62 and HCD-induced insulin resistance and dysregulated glucose handling. Mean HOMA-IR values ± SEM were determined from serum fasting glucose and insulin levels at cull for male (a) and female (b) mice. Measurement of fasting glucose (c, d) and glucose tolerance (GTT-AUC, e-f) were undertaken one week before cull days and presented as mean values ± SEM of individual male (c, e, g) and female (d, f, h) mice. The time course for glucose clearance for the d500 cohorts is presented (g, h) with data expressed as the mean blood glucose levels ± SEM of individual mice. Cohort sizes: males: chow—d56, n = 6 (glucose, GTT-AUC, HOMA-IR); d160 and d340, n = 6; d500, n = 5 (glucose; GTT-AUC), 4 (HOMA-IR); HCD-PBS—d160, n = 9 (HOMA-IR), 10 (glucose; GTT-AUC); d340, n = 11 (glucose, GTT-AUC), 9 (HOMA-IR); d500, n = 6 (glucose, GTT-AUC, HOMA-IR); HCD-ES-62—d160, n = 9 (HOMA-IR), 10 (glucose; GTT-AUC); d340, n = 12 (glucose, GTT-AUC), 9 (HOMA-IR); d500, n = 6 (glucose, GTT-AUC, HOMA-IR) and females: chow—d56, n = 6; d160 and d340, n = 5; d500, n = 3; HCD-PBS—d160, n = 9; d340, n = 9 (HOMA-IR), 11 (glucose, GTT-AUC); d500, n = 6 (glucose, GTT-AUC, HOMA-IR); HCD-ES-62—d160, n = 9 (HOMA-IR), 10 (glucose, GTT-AUC); d340, n = 9 (HOMA-IR), 12 (glucose, GTT-AUC); d500, n = 6 (glucose, GTT-AUC, HOMA-IR). For clarity, only significant differences between the HCD-PBS and HCD-ES-62 cohorts are shown on the figures, where significance is denoted by **p < 0.01.
(EPS)

S7 Fig ES-62 and liver steatosis and dysfunctional mitochondrial function in HCD-aged mice. Representative images (scale bar 100 μm) of liver from male (a) and female (b) chow- and HCD- (PBS- or ES-62-treated) mice stained with H & E and resultant quantitative analysis of fat deposition where data are presented as the mean values ± SEM where n = 5–6 individual male (c) and female (d) mice at each time point and the values for each mouse are means derived from n = 3 replicate analyses. Mitochondrial respiration (oxygen consumption rate, OCR) was measured in livers from male (e, g, i) and female (f, h, j) chow- or HCD-mice at d160 (e, f), d340 (g, h) and d500 (i, j). OCR was measured under Basal (substrate alone), State 3 (ADP), State 4 (oligomycin), State 3u (FCCP) and non-mitochondrial (antimycin A plus rotenone; Anti-A) conditions. Data are presented as the values ± SEM of individual mice where cohort sizes were: male, d160—chow n = 6; HCD-PBS n = 10; HCD-ES-62 n = 9; d340—chow n = 6, HCD-PBS n = 7, HCD-ES-62, n = 8; d500 chow—n = 4, HCD-PBS, n = 4; HCD-ES-62, n = 4; female, d160—chow n = 5; HCD-PBS n = 9; HCD-ES-62 n = 10 d340—chow n = 6; HCD-PBS, n = 11; HCD-ES-62, n = 12; d500 –chow n = 3, HCD-PBS n = 4, HCD-ES-62, n = 4. For clarity, only significant differences between the HCD-PBS and HCD-ES-62 cohorts are shown on the figures, where significance is denoted by *p < 0.05 and **p < 0.01.
(TIFF)

S8 Fig ES-62 and REDOX status in HCD-fed mice. RT-qPCR analysis of cytochrome C (a, b) and HMOX-1 (c, d) expression in liver from male (a, c) and female (b, d) chow- and HCD- (PBS- or ES-62-treated) mice are shown where data are expressed as mean 2^ΔCT values ± SEM of individual mice and the values for each mouse are means of n = 3 replicate analyses. Male cohort sizes: chow—d56, n = 6; d160, n = 6; d340, n = 6; d500, n = 5; HCD-PBS—d160, n = 10; d340, n = 11; d500, n = 6; HCD-ES-62—d160, n = 10; d340, n = 12; d500, n = 6 and female cohort sizes: chow—d56, n = 6; d160, n = 6; d340, n = 6; d500, n = 5; HCD-PBS—d160, n = 5; d340, n = 11; d500, n = 6; HCD-ES-62—d160, n = 5; d340, n = 12; d500, n = 6. Determination of superoxide dismutase (SOD; e, f) and protein carbonylation (g, h) activities in liver from male (e, g) and female (f, h) chow- and HCD- (PBS- or ES-62-treated) mice where data are expressed as mean activity values ± SEM of individual mice. Male cohort sizes: chow—d56, n = 6 (SOD), n = 3 (protein carbonylation); d160, n = 6 (SOD), n = 3 (protein carbonylation); d340, n = 6 (SOD), n = 3 (protein carbonylation); d500, n = 5; HCD-PBS—d160, n = 10 (SOD), n = 6 (protein carbonylation); d340, n = 10 (SOD), n = 11 (protein carbonylation); d500, n = 6; HCD-ES-62—d160, n = 8 (SOD), n = 6 (protein carbonylation); d340, n = 10 (SOD), n = 12 (protein carbonylation); d500, n = 6 and female cohort sizes: chow—d56, n = 5 (SOD), n = 3 (protein carbonylation); d160, n = 6 (SOD), n = 3 (protein carbonylation); d340, n = 6 (SOD), 3 (protein carbonylation); d500, n = 4; HCD-PBS—d160, n = 9 (SOD), n = 6 (protein carbonylation); d340, n = 10 (SOD), n = 9 (protein carbonylation); d500, n = 6; HCD-ES-62—d160, n = 10 (SOD), n = 6 (protein carbonylation); d340, n = 10 (SOD), n = 11 (protein carbonylation); d500, n = 6. For clarity, only significant differences between the HCD-PBS and HCD-ES-62 cohorts are shown on the figures, where significance is denoted by *p < 0.05, **p < 0.01 and ***p < 0.001.
(EPS)

S9 Fig ES-62 modulates HCD-induced inflammation in the liver. RT-qPCR analysis of IL-1β, IL-18 and NLRP3 expression in liver from male (a, c, e) and female (b, d, f) chow- and HCD- (PBS- or ES-62-treated) mice where data are expressed as mean 2^ΔCT values ± SEM of individual mice and the values for each mouse are means of n = 3 replicate analyses. Male cohort sizes: chow—d56, n = 6; d160, n = 6; d340, n = 6; d500, n = 5; HCD-PBS—d160, n = 10; d340, n = 11; d500, n = 6; HCD-ES-62—d160, n = 10; d340, n = 12; d500, n = 6 and female cohort sizes: chow—d56, n = 6; d160, n = 6; d340, n = 6; d500, n = 5; HCD-PBS—d160, n = 5; d340, n = 11; d500, n = 6; HCD-ES-62—d160, n = 5; d340, n = 12; d500, n = 6. For clarity, only significant differences between the HCD-PBS and HCD-ES-62 cohorts are shown on the figures, where significance is denoted by *p < 0.05, **p < 0.01 and ***p < 0.001.
(EPS)

S10 Fig ES-62 and HCD-accelerated gut damage during ageing. Images of ileum and colon tissue from male and female chow- and HCD- (PBS- or ES-62-treated) mice (see Fig 5 for representative images) stained with H & E (to visualise basal lamina) and Gömöri’s Trichrome (to visualise collagen deposition) were analysed quantitatively for basal lamina width (ileum, a, b; colon, e, f) and collagen deposition (ileum, c, d; colon, g, h). Data are presented as the mean values ± SEM where n = 4–6 individual male (a, c, e, g) and female (b, d, f, h) mice from each group at each time point and the values for each mouse are means derived from n = 3 replicate analyses. Goblet cell loss (i, j) was scored from PAS-stained sections of ileum (i) and colon (j) and data presented as the mean values, where n = 5–6 individual male and female mice from each group at the d500 time point and the values for each mouse are means derived from n = 3 replicate analyses.
(EPS)

S11 Fig Gating strategies. (a) Following exclusion of dead cells/cell debris and gating of fat cells (by forward scatter versus side scatter), cell doublets were excluded prior to subsequent gating of SiglecF+ and F4/80 populations (Fig 3 and S3 Fig) relative to the relevant isotype and FMO controls. Splenocytes were gated for singlets (FSC-H vs. FSC-A), morphology (FSC-A vs. SSC-A) and then live cells determined by their uptake of the fixable live/dead cell stain before gating prior to assessing expression of (b) CD19^+^CD21^-^CD23^-^CD11c^+^ B cells (Fig 6) or (c) CD19^+^ IL-10^+^ B cells (Fig 7) with reference to relevant isotype controls.
(TIFF)
