# Supplementary material for: The parasitic worm product ES-62 promotes health- and life-span in a high calorie diet-accelerated mouse model of ageing
Source: PLoS Pathog. 2020 Mar 12;16(3):e1008391. doi: 10.1371/journal.ppat.1008391 (PMC7108737; doi:10.1371/journal.ppat.1008391)
Supplement: S1 Table — aCox regression analysis of PBS- and ES-62 -treated mice. Age at death was analysed in pooled male and female PBS and ES-62 mice using Cox regression analysis (SPSS Statistics v22). The independent variables, Treatment (PBS or ES-62) and Sex (male or female), were replaced with a set of indicator variables to denote the presence or absence of category membership. B is the un-standardised regression coefficient and its standard error is SE, its Wald test statistic value, Wald, the degrees of freedom, df and the significance, Sig. Exp(B) for the covariate of interest is the predicted change in the hazard ratio for a unit increase in the predictor. Sample size = 48 for PBS- and 48 for ES-62-treated mice. bDescriptive statistics of this longevity study were derived using the GraphPad Prism8 logrank test survival software package. (PDF) [file ppat.1008391.s001.pdf]

S1 Table: Descriptive Statistics of the longevity cohort

Cox Regression Analysis[a]						
**Covariate**	**B**	**SE**	**Wald**	**Df**	**Sig**	**Exp(B)**
Treatment	-0.130	0.206	0.399	1	0.528	0.878
Sex	0.541	0.222	5.933	1	0.015	1.717

Descriptive Statistics[b]						
**Sex**	**Treatment**	**Median**	**Mean**	**Min-Max**	**Oldest 10%**	**n**
Male	PBS	629	636 ± 26.7	222-869	782	24
	ES-62	703	676 ± 29.8	193-869	821	24
Female	PBS	653	637 ± 16.7	443 -760	727	24
	ES-62	629	610 ± 23.9	366 -779	745	24
