# Supplementary material for: The parasitic worm product ES-62 promotes health- and life-span in a high calorie diet-accelerated mouse model of ageing
Source: PLoS Pathog. 2020 Mar 12;16(3):e1008391. doi: 10.1371/journal.ppat.1008391 (PMC7108737; doi:10.1371/journal.ppat.1008391)
Supplement: S2 Table — Individual mice (each cohort n = 24) in the lifespan study were monitored daily, weighed twice-weekly and analysed for grip strength monthly, but otherwise left undisturbed until they died (natural death). If death appeared imminent (as assessed by our humane end-points) mice were weighed, euthanised and examined for macroscopic pathological changes using previously described protocols[82,83] with this date considered date of death. The numbers of mice displaying one or more pathologies post mortem as well those undergoing natural death or euthanasia in each cohort is shown. (PDF) [file ppat.1008391.s002.pdf]

S2 Table: Pathology identified post-mortem in mice from the lifespan cohort

**Pathology**	**Male**		**Female**	
	**PBS**	**ES-62**	**PBS**	**ES-62**
GI tract obstruction/tumour	1	1	2	1
Hepatic tumour	11	10	10	10
Gonadal tumour	-	-	2	-
Multi-organ tumour	1	-	-	1
Musculoskeletal pathology	-	-	1	2
Non-healing dermatitis	-	-	1	-
Infection	-	-	-	1
None identified	11	14	10	9
**Natural death**	6	3	2	4
**Euthanasia**	18	21	22	20
