# Supplementary figures and images for: The parasitic worm product ES-62 promotes health- and life-span in a high calorie diet-accelerated mouse model of ageing
Source: PLoS Pathog. 2020 Mar 12;16(3):e1008391. doi: 10.1371/journal.ppat.1008391 (PMC7108737; doi:10.1371/journal.ppat.1008391)

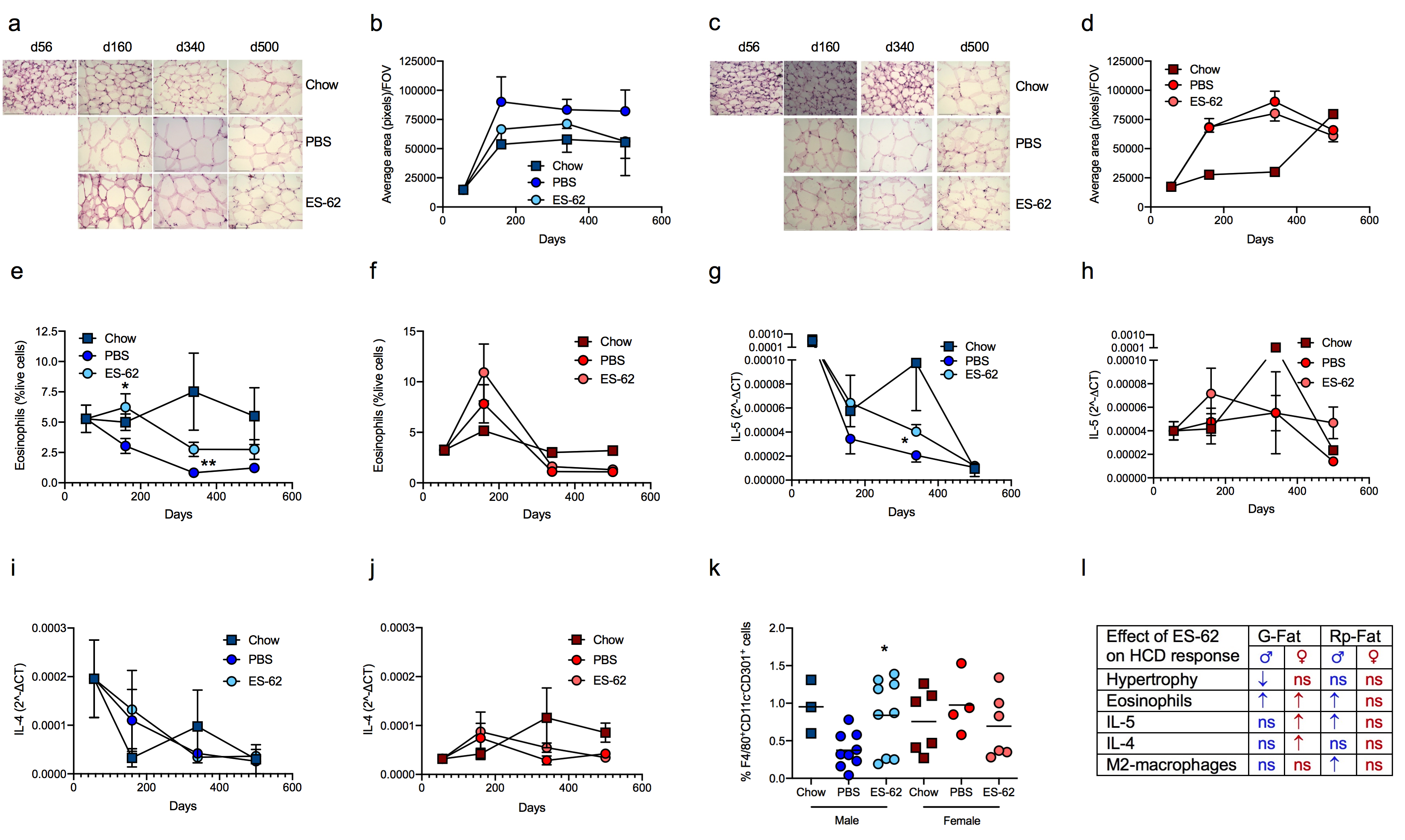

Supplement: S3 Fig — Representative images (scale bar 100 μm) of retroperitoneal fat from male (a) and female (c) chow- and HCD- (PBS- or ES-62-treated) mice stained with H & E and resultant quantitative analysis of adipocyte size where data are presented as the mean values ± SEM of individual male (b) and female (d) mice and the values for each mouse are derived from n = 3 replicate analyses. Levels of eosinophils in retroperitoneal fat are presented as the mean values ± SEM at each time point where male (e) cohort sizes are: chow—d56, n = 5; d160, n = 3; d340, n = 5; d500, n = 5; HCD-PBS—d160, n = 9; d340, n = 10; d500, n = 6; HCD-ES-62—d160, n = 9; d340, n = 12; d500, n = 6 and female (f) cohort sizes are: chow—d56, n = 6; d160, n = 6; d340, n = 6; d500, n = 5; HCD-PBS—d160, n = 4; d340, n = 11; d500, n = 6; HCD-ES-62—d160, n = 6; d340, n = 12; d500, n = 6. qRT-PCR analysis of IL-5 and IL-4 mRNA expression in retroperitoneal fat from chow- and HCD-fed (PBS- or ES-62-treated) mice is presented (g-j) as mean 2^ΔCT values ± SEM of individual mice and the values for each mouse are means of n = 3 replicate analyses. Male cohort sizes: chow—d56, n = 5; d160, n = 3; d340, n = 4; d500, n = 2; HCD-PBS—d160, n = 10; d340, n = 11; d500, n = 6; HCD-ES-62—d160, n = 8; d340, n = 11; d500, n = 6. Female cohort sizes: chow—d56, n = 5; d160, n = 4; d340, n = 5; d500, n = 2; HCD-PBS—d160, n = 7; d340, n = 9; d500, n = 6; HCD-ES-62—d160, n = 9; d340, n = 11; d500, n = 6. The levels of F4/80+CD11c-CD301+ cells (k) in gonadal fat in male and female chow- and HCD- (PBS- or ES-62-treated) mice in the d160 cohorts are shown. For clarity, only significant differences between the HCD-PBS and HCD-ES-62 cohorts are shown on the figures, where significance is denoted by *p < 0.05 and ***p < 0.001. However, in (b) the chow cohort are significantly different (p<0.05) from the HCD-PBS, but not the HCD-ES-62 mice. (l) Summary of the disconnect between adipocyte health and type-2 responses in ES-62-treated HCD mice i [file ppat.1008391.s005.tiff]

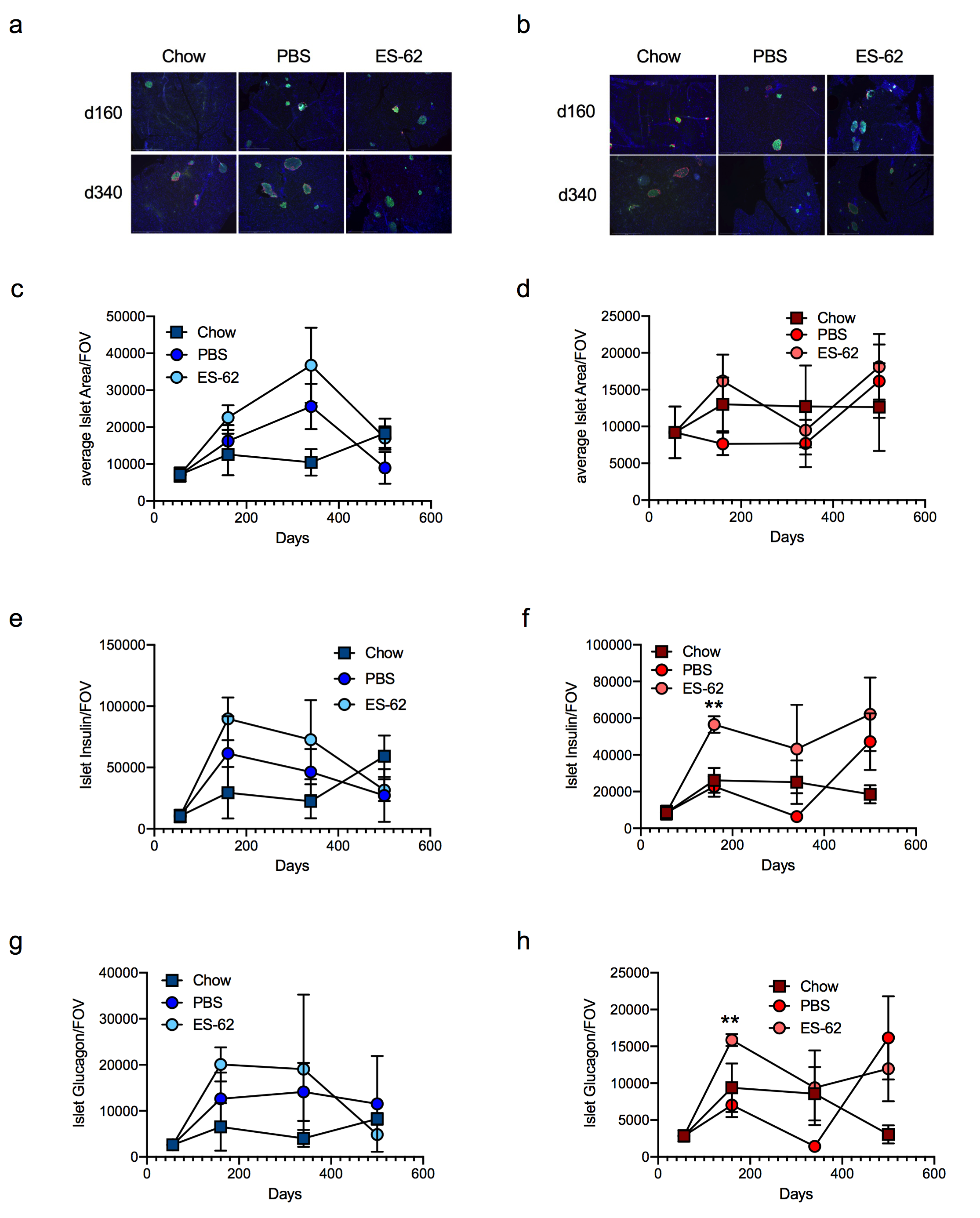

Supplement: S5 Fig — Representative images (scale bar 500 μm) of pancreas at d160 and d340 from male (a) and female (b) mice stained for insulin (green), glucagon (red) and counterstained with DAPI (blue). Quantitative analysis of islet size (c, d) and production of insulin (e, f) and glucagon (g, h) where data are presented as the mean values (of triplicate analyses) ± SEM of individual male (c, e, g) and female (d, f, h) mice (n = 4–6) at each time-point. For clarity, only significant differences between the HCD-PBS and HCD-ES-62 cohorts are shown on the figures, where significance is denoted by **p < 0.01. (TIFF) [file ppat.1008391.s007.tiff]

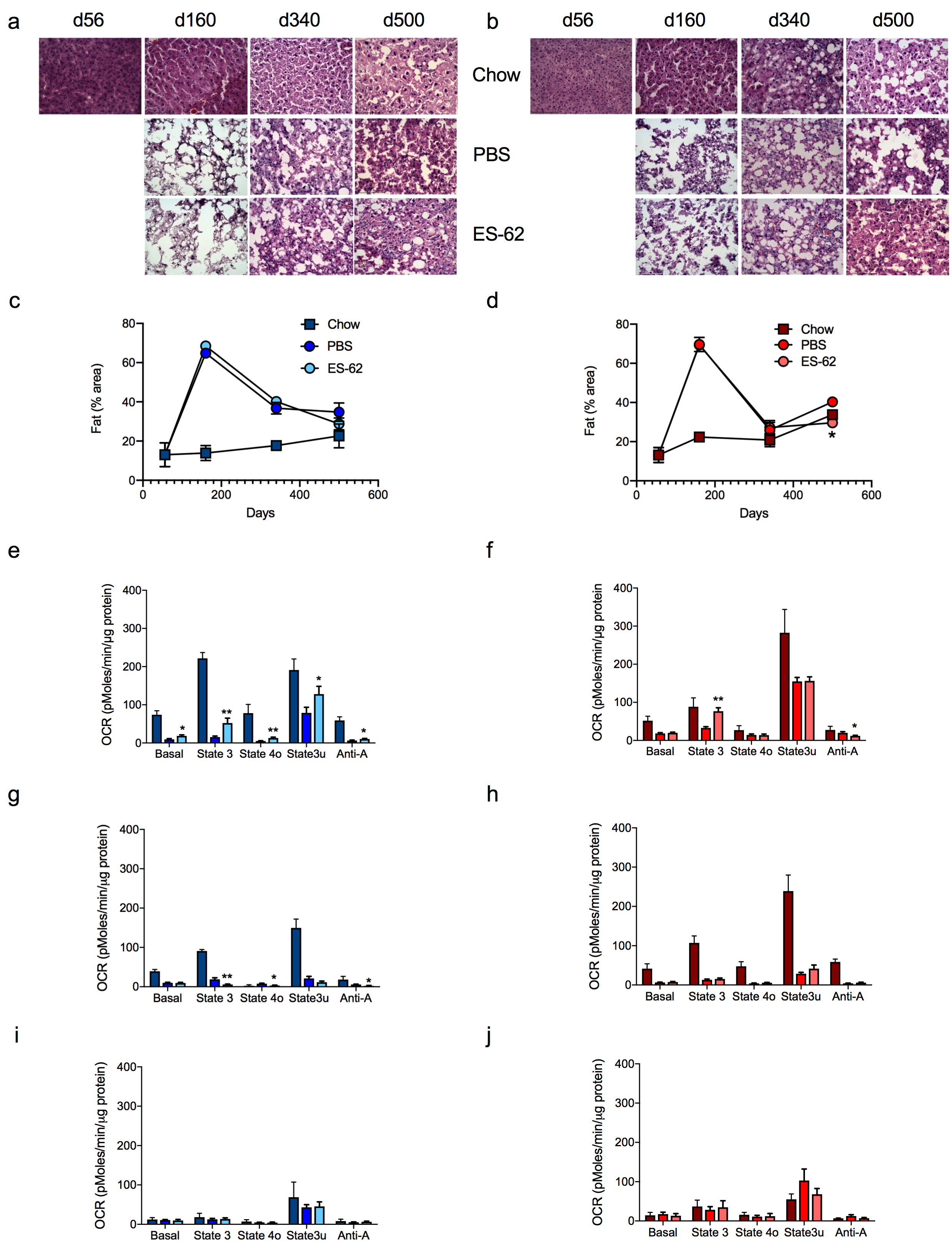

Supplement: S7 Fig — Representative images (scale bar 100 μm) of liver from male (a) and female (b) chow- and HCD- (PBS- or ES-62-treated) mice stained with H & E and resultant quantitative analysis of fat deposition where data are presented as the mean values ± SEM where n = 5–6 individual male (c) and female (d) mice at each time point and the values for each mouse are means derived from n = 3 replicate analyses. Mitochondrial respiration (oxygen consumption rate, OCR) was measured in livers from male (e, g, i) and female (f, h, j) chow- or HCD-mice at d160 (e, f), d340 (g, h) and d500 (i, j). OCR was measured under Basal (substrate alone), State 3 (ADP), State 4 (oligomycin), State 3u (FCCP) and non-mitochondrial (antimycin A plus rotenone; Anti-A) conditions. Data are presented as the values ± SEM of individual mice where cohort sizes were: male, d160—chow n = 6; HCD-PBS n = 10; HCD-ES-62 n = 9; d340—chow n = 6, HCD-PBS n = 7, HCD-ES-62, n = 8; d500 chow—n = 4, HCD-PBS, n = 4; HCD-ES-62, n = 4; female, d160—chow n = 5; HCD-PBS n = 9; HCD-ES-62 n = 10 d340—chow n = 6; HCD-PBS, n = 11; HCD-ES-62, n = 12; d500 –chow n = 3, HCD-PBS n = 4, HCD-ES-62, n = 4. For clarity, only significant differences between the HCD-PBS and HCD-ES-62 cohorts are shown on the figures, where significance is denoted by *p < 0.05 and **p < 0.01. (TIFF) [file ppat.1008391.s009.tiff]

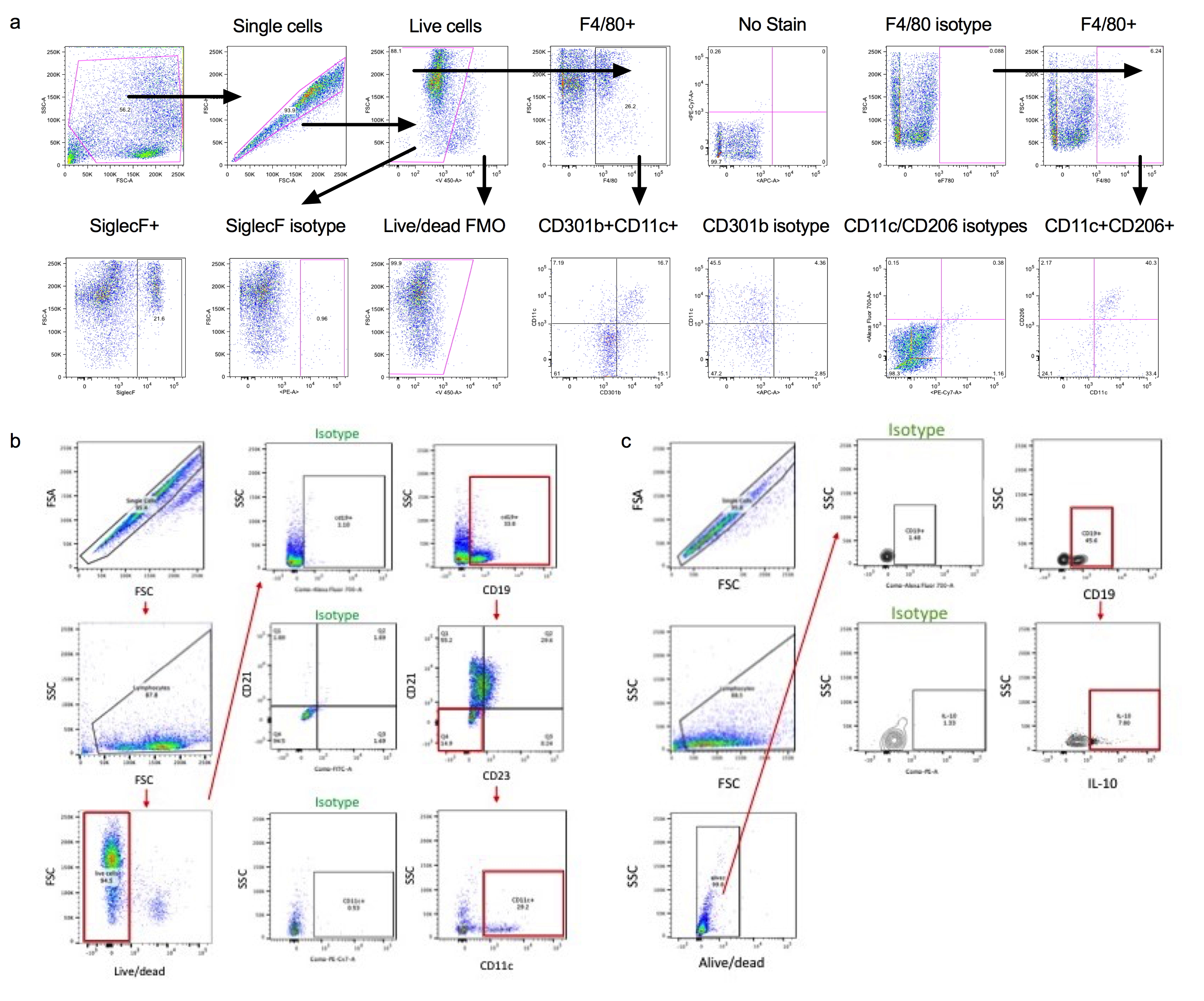

Supplement: S11 Fig — (a) Following exclusion of dead cells/cell debris and gating of fat cells (by forward scatter versus side scatter), cell doublets were excluded prior to subsequent gating of SiglecF+ and F4/80 populations (Fig 3 and S3 Fig) relative to the relevant isotype and FMO controls. Splenocytes were gated for singlets (FSC-H vs. FSC-A), morphology (FSC-A vs. SSC-A) and then live cells determined by their uptake of the fixable live/dead cell stain before gating prior to assessing expression of (b) CD19+CD21-CD23-CD11c+ B cells (Fig 6) or (c) CD19+ IL-10+ B cells (Fig 7) with reference to relevant isotype controls. (TIFF) [file ppat.1008391.s013.tiff]
